# Association of all-cause mortality with sugar intake from different sources in the prospective cohort of UK Biobank participants

**DOI:** 10.1017/S0007114522003233

**Published:** 2023-07-28

**Authors:** Anna Kaiser, Sylva M. Schaefer, Inken Behrendt, Gerrit Eichner, Mathias Fasshauer

**Affiliations:** 1 Institute of Nutritional Science, Justus-Liebig University of Giessen, Giessen, 35390, Germany; 2 Mathematical Institute, Justus-Liebig University of Giessen, Giessen, Germany; 3 Department of Internal Medicine (Endocrinology, Nephrology, and Rheumatology), University of Leipzig, Leipzig, Germany

**Keywords:** Carbohydrates, Mortality, Prospective cohort study, Sugar, UK Biobank

## Abstract

The present study elucidates the association of intrinsic sugars and free sugars (FS) from all relevant sources with all-cause mortality in the prospective UK Biobank cohort. Sugar intake was assessed in 186 811 UK Biobank participants who completed at least one web-based 24-h dietary recall (Oxford WebQ). Cox proportional hazard regression models for all-cause mortality were used with sugar intake from different sources included as penalised cubic splines to allow non-linear predictor effects. Over a mean follow-up of 12·3 years, 8576 (4·6 %) deaths occurred. FS but not intrinsic sugars were significantly and dose-dependently associated with hazard ratio (HR) for all-cause mortality. The association with all-cause mortality was significant and dose dependent for FS in beverages, but not in solids with the mean (CI) HR at 50 g/d *v*. 0 g/d consumption at 1·10, 95 % CI (1·07, 1·14) and 1·01, 95 % CI (0·98, 1·03), respectively. Within the beverages subcategories, a significant dose-dependent association with mortality was detected for FS in soda/fruit drinks and milk-based drinks whereas this relation was NS for FS in pure juice and tea/coffee. FS in four different subtypes of solids, i.e. treats, cereals, toppings and sauces, were not positively associated with all-cause mortality. Major findings were robust in sensitivity analyses. In conclusion, only some FS sources were associated with all-cause mortality. Interventions targeting FS subtypes might be most effective concerning mortality if focused on the reduction of soda/fruit drinks and milk-based sugary drinks; however, the present results need to be confirmed by independent studies.

Besides a sedentary lifestyle, unhealthy eating patterns are major contributors to body weight gain and associated disease states including hypertension, impaired glucose control and CVD^(1–4)^. To combat obesity and its sequelae, various dietary interventions have focused on macronutrient composition with low-carbohydrate diets being one popular approach^(5,6)^. High-quality evidence suggests that the reduction of carbohydrates leads to significant short-term weight loss and metabolic improvements^(5,6)^. However, a broad range of food items has to be excluded from the diet that limits the diversity of choices contributing to poor long-term adherence and weight regain^(7)^. Furthermore, carbohydrates with beneficial effects might also be excluded from the diet, e.g. complex carbohydrates present in whole grains and legumes^(1)^.

Therefore, rather than reducing total carbohydrates, more recent interventions have focused on specific carbohydrate subtypes, particularly sugars^(8,9)^. Sugars are all mono- and disaccharides^(10)^, and different sugar sources relevant to the present study are summarised in Supplementary Fig. 1. According to the WHO, sugars can be divided into free sugars (FS) and intrinsic sugars^(11)^. FS are all monosaccharides and disaccharides added to foods by the manufacturer, cook or consumer, plus sugars naturally present in honey, syrups and fruit juices^(11)^. Since a clear link between FS consumption and body weight gain exists, the WHO recommends to limit FS throughout the life course to < 10 % of total energy intake, i.e. 50 g FS/d for a 2000 kcal diet, and optimally to even below 5 %^(11)^. The National Health Service (NHS) England also recommends a consumption of no more than 5 % of total energy intake from FS^(12)^. However, these recommendations^(11,12)^ do not differentiate between FS sources like FS from beverages or solids. Within beverages, FS are present in soda/fruit drinks, pure juice, milk-based drinks and tea/coffee. FS in solids can originate from treats, cereals, toppings and sauces. Intrinsic sugars represent all sugars that are not FS including sugars from fruit, vegetables and lactose in dairy products^(11)^. In contrast to FS, intrinsic sugars are not associated with adverse metabolic and cardiovascular effects in several studies^(11)^. However, no study so far has elucidated the link between intrinsic sugars and mortality.

Taking published evidence into consideration, it has been well established that FS promote metabolic and CVD^(8,13–18)^. However, no study so far has systematically assessed the association of FS from all relevant sources with all-cause mortality. To address this open point, all major FS sources were assessed within the current study including beverages, solids and their subtypes. All analyses were conducted in a large, well-characterised population of 186 811 UK Biobank participants using penalised cubic splines to allow, in particular, non-linear predictor effects. We hypothesised that the association between FS and mortality depends on FS source with adverse effects being especially related to beverages and differential associations seen for specific beverage subtypes. Furthermore, we hypothesised that high consumption of intrinsic sugars is not related to all-cause mortality.

## Methods

### Study and participants

All analyses are based on the UK Biobank study that recruited more than 500 000 participants between 2006 and 2010 at twenty-two assessment centres across the UK^(19)^. All participants were assessed at baseline via a self-completed touchscreen questionnaire, a personal interview and physical measurements^(19)^. Participants for the current study were selected from the UK Biobank cohort as presented in Supplementary Fig. 2. Similar to a previous study^(20)^, the following exclusion criteria were applied: (1) malabsorption/coeliac disease; (2) missing lifestyle risk factors (physical activity and/or smoking status); (3) missing socio-economic factors (Townsend deprivation index, total household income, ethnic background, highest qualification and/or overall health rating); (4) missing data of the physical exam (BMI, systolic blood pressure); (5) history of diabetes mellitus and (6) implausible energy or carbohydrate intake, i.e. being in the upper 0·1 % of total energy and/or carbohydrate intake or total energy intake <1·1 × basal metabolic rate – 500 kcal (under-reporting) or >2·5 × basal metabolic rate + 500 kcal (over-reporting) resulting in a study population of 186 811 participants (online Supplementary Fig. 2 and 3). Basal metabolic rate was calculated according to the Oxford equation^(21)^.The UK Biobank study was approved by the North West Multicentre Research Ethics Committee, and written informed consent was provided by all participants at baseline^(19)^.

### Exposure assessment

To provide detailed dietary information, a web-based 24-h dietary recall (Oxford WebQ) was completed that assesses consumption of 206 foods and thirty-two beverages^(22)^. The Oxford WebQ was specifically developed for use in large population studies, and completing a single questionnaire has been validated against an interviewer-administered 24-h dietary recall^(23)^. A further validation study using objective biomarkers indicates that the results of the Oxford WebQ are broadly similar to those obtained by more researcher-intensive and expensive 24-h recall delivered and coded by trained researchers^(24)^. Using the mean of two to five, repeat administrations substantially improve measurement properties^(24)^.

UK Biobank participants could complete the Oxford WebQ on up to five occasions. For participants who filled out more than one questionnaire, the mean dietary intake was used for all primary and sensitivity analyses except when only the first completed Oxford WebQ was considered (online Supplementary Fig. 11). The five occasions for participants to fill out the Oxford WebQ were April 2009 to September 2010, February 2011 to April 2011, June 2011 to September 2011, October 2011 to December 2011 and April 2012 to June 2012^(25)^. Within these periods, participants could complete the questionnaire for weekdays or weekends. Intake of sugar and its subtypes was estimated with methodology similar to a recent study^(26)^. In brief, for each item of the Oxford WebQ, energy and total sugar values were estimated based on McCance and Widdowson’s The Composition of Foods and its supplements as suggested by Liu and co-workers^(23)^, the UK Data Archive Standard Recipes Database^(27)^ and product labels. FS were defined similar to Wanselius and co-workers^(28)^, and the decision procedure is presented in Supplementary Table 1. Standard portion sizes were taken from the UK Food Standards Agency^(29)^ and product labels. In the Oxford WebQ, participants specify the number of standard portions consumed of specific food items with quarter and half portions available for some items, e.g. cereal bars and sweet biscuits. For each participant, average intake (g/d) of the sugar subtype under study was calculated by multiplying the frequency of each food item by the estimated content of this sugar subtype in that item in a standard portion. Intrinsic sugars were calculated as the difference between total sugars and FS.

### Outcome assessment

Mortality data with date of death were provided by the NHS Information Centre for participants from England and Wales and by the NHS Central Register, Scotland for participants from Scotland^(30)^. Follow-up time was defined as duration between baseline assessment and date of death or censoring (12 November 2021), whichever came first.

### Statistical analyses

Data were imported, processed, analysed and graphically displayed with R version 4.0.5^(31)^ as recently described by our group^(32,33)^. Cox proportional hazard regression models of overall survival time were fitted with sugar subtypes and energy intake included as penalised cubic splines with their degrees of freedom set to 4. Cubic splines instead of discretised ordinal predictors, e.g. cut-off values, were used for analyses of sugar subtypes to allow continuous non-linear predictor effects. Besides energy intake, models were adjusted for age (quintiles), sex (female, male), ethnic background (Caucasian, Group composed of Mixed, Asian, Black, Chinese and other), BMI (<18·5, 18·5 to <25, 25 to <30 and ≥30 kg/m^2^), systolic blood pressure (quintiles), Townsend deprivation index (quintiles), general health status (poor, fair, good and excellent), total household income (<18, 18 to <31, 31 to <52, 52 to <100 and ≥100 k£, unknown), highest qualification (none of the below, national exams at age 16 years, vocational qualifications or optional national exams at ages 17–18 years, professional, College or University), smoking status (never, previous, current occasional, current <10, 10 to 14, 15 to 19 and ≥20 cigarettes/d), alcohol intake (<1, 1 to <8 and 8 to <16, ≥16 g/d), physical activity (metabolic equivalent of task (MET)-min/week derived from the Oxford WebQ; quintiles) and history of psychiatric disease (yes, no). The proportional-hazard assumption was tested based on scaled Schoenfeld residuals, and all covariates violating this assumption after Holm adjustment for multiple testing were stratified in the final models.

In all analyses, the nadir was defined as the consumption of specific sugar subtypes with the lowest estimated hazard ratio (HR) over the range from zero to the 99 %-quantile of consumption and the HR at the nadir was set to 1 to simplify presentations and comparisons. Mean HR with pointwise 95 % CI are shown for all mortality analyses and are also described in the text for defined levels of sugar subtype intake.

The analysis of each penalised cubic spline is segregated into its linear and nonlinear effects whose significances are documented by the respective *P* values (*P* ^lin^ for the linear and *P* ^non-lin^ for the nonlinear effect) of Wald-type tests for joint significance of the multiple coefficients associated with the respective linear or nonlinear portion of the penalised spline fit^(34,35)^. If both *P* ^lin^ and *P* ^non-lin^ were non-significant, no further interpretation of the nadir or other individual HR was performed.

Various sensitivity analyses were performed similar as described by Anderson and co-workers^(36)^. Three analyses were run to check for reverse causation. First, participants lost to follow-up or dying within 2 years after baseline were excluded (landmark analysis). Second, participants who indicated at baseline that they had lost weight unintentionally were excluded. Third, participants with a history of cardiovascular disease and cancer were removed to assess whether prevalence of these diseases at baseline affected the findings. To control for unrepresentative consumption data, participants who reported their previous day´s diet as non-typical and who completed the Oxford WebQ for a weekend day, respectively, on at least one occasion were excluded in two further sensitivity analyses. Participants with only one or up to two Oxford WebQ were removed from the analysis to address potential variation, i.e. low reproducibility, in sugar intake based on a single and up to two Oxford WebQ, respectively. To consider intake most adjacent to baseline assessment, analyses were re-run using only the first Oxford WebQ questionnaire. Waist:hip ratio (WHR) and height instead of BMI were used as alternative measures for body composition. A diet quality score was generated combining five dietary components, i.e. fat, fruit, vegetables, red meat and processed meat consumption as described^(36)^ to further control for residual confounding by dietary factors. History of psychiatric disease was not included as a covariate in another sensitivity analysis to assess whether exclusion of this parameter would substantially affect the main Cox model results. A *P* value of < 0·05 was considered statistically significant in all analyses.

## Results

### Baseline characteristics and deaths in UK Biobank participants

Baseline data of the study population in total and depending on quintiles of FS intake are summarised in Table 1. Mean (standard deviation (sd)) age of the study population was 56 (8) years with 57·2 % of participants being female. Over a mean (sd) follow-up of 12·3 (1·4) years and 2·3 million person-years, 3811 deaths occurred in females and 4765 in males, i.e. a total of 8576 deaths. The mean (sd) number of dietary questionnaires per participant was 2·2 (1·2).

**Table 1.** Baseline characteristics of the UK Biobank cohort*

Parameters			Quintiles of FS intake (g/d)									
	Total cohort (*n* 186 811)		0·0 to 32·7 (*n* 37 362)		32·7 to 48·8 (*n* 37 362)		48·8 to 65·4 (*n* 37 362)		65·4 to 88·6 (*n* 37 362)		88·6 to 461·4 (*n* 37 363)	
	*n* or mean	% or SD	*n* or mean	% or SD	*n* or mean	% or SD	*n* or mean	% or SD	*n* or mean	% or SD	*n* or mean	% or SD
Age (years)	56	8	56	8	56	8	56	8	56	8	55	8
Total physical activity (MET-min/week)	4130	2651	4026	2619	4049	2518	4068	2521	4156	2622	4349	2940
Townsend index	−1·6	2·8	−1·5	2·9	−1·7	2·8	−1·7	2·8	−1·8	2·8	−1·6	2·9
BMI (kg/m^2^)	26·6	4·3	26·6	4·3	26·5	4·3	26·5	4·3	26·5	4·3	26·9	4·5
SBP (mmHg)	139	19	138	20	138	20	139	19	139	19	139	19
Women	106 901	57·2	24 454	65·5	23 501	62·9	21 929	58·7	20 162	54·0	16 855	45·1
Smoking status												
Never	107 455	57·5	19 747	52·9	21 046	56·3	21 909	58·6	22 336	59·8	22 417	60·0
Previous	65 950	35·3	14 630	39·2	13 835	37·0	13 135	35·2	12 545	33·6	11 805	31·6
Current	13 406	7·2	2985	8·0	2481	6·6	2318	6·2	2481	6·6	3141	8·4
Total household income/year (k£)												
<18	25 016	13·4	4759	12·7	4685	12·5	4799	12·8	4988	13·4	5785	15·5
18 to <100	131 045	70·2	26 015	69·6	26 295	70·4	26 349	70·5	26 380	70·6	26 006	69·6
≥100	12 448	6·7	2815	7·5	2697	7·2	2593	6·9	2331	6·2	2012	5·4
Unknown	18 302	9·8	3773	10·1	3685	9·9	3621	9·7	3663	9·8	3560	9·5
Ethnic background												
Caucasian	180 058	96·4	36 024	96·4	36 177	96·8	36 166	96·8	36 097	96·6	35 594	95·3
Highest qualification												
None of the below	15 007	8·0	3336	8·9	3004	8·0	2765	7·4	2826	7·6	3076	8·2
National exams at age 16 to 18 years	61 183	32·8	12 760	34·2	12 049	32·2	11 756	31·5	11 947	32·0	12 671	33·9
Professional	28 993	15·5	5506	14·7	5684	15·2	5873	15·7	5890	15·8	6040	16·2
College or University	81 628	43·7	15 760	42·2	16 625	44·5	16 968	45·4	16 699	44·7	15 576	41·7
Overall health rating												
Poor and fair	34 488	18·5	6644	17·8	6261	16·8	6391	17·1	6867	18·4	8325	22·3
Good	113 284	60·6	22 618	60·5	22 909	61·3	22 928	61·4	22 783	61·0	22 046	59·0
Excellent	39 039	20·9	8100	21·7	8192	21·9	8043	21·5	7712	20·6	6992	18·7
History of psychiatric disease	12 290	6·6	2417	6·5	2320	6·2	2234	6·0	2482	6·6	2837	7·6
Number of Oxford WebQ	2·2	1·2	2·0	1·1	2·3	1·2	2·4	1·2	2·3	1·2	2·1	1·1

FS, free sugar; MET, metabolic equivalent of task; SBP, systolic blood pressure.

* Categorical variables are presented as number (percentage) and continuous variables as mean (sd).

### Free sugars *v.* intrinsic sugars and all-cause mortality

**Fig. 1.**
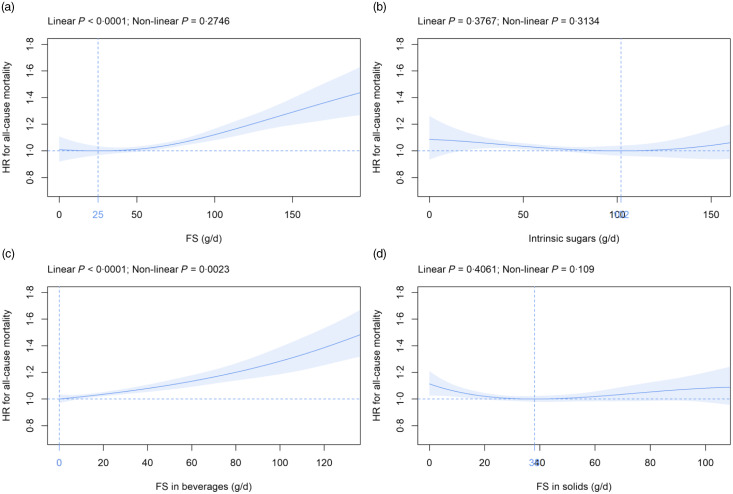
Association of (a) FS, (b) intrinsic sugars, (c) FS in beverages and (d) FS in solids intake (all g/d) with all-cause mortality. Models are adjusted for energy intake, age, sex, ethnic background, BMI, systolic blood pressure, Townsend deprivation index, general health status, total household income, highest qualification, smoking status, alcohol intake, physical activity and history of psychiatric disease as summarised in the Methods section. Covariates not fulfilling the proportional hazard assumption are stratified. The nadir is indicated in blue. FS, free sugars; HR, hazard ratio.

Mean (sd) intake of FS and intrinsic sugars was 63·0 (37·4) and 66·9 (29·8) g/d, respectively (Table 2). FS intake was beyond the 5 % and 10 % thresholds of total energy recommended by the NHS England^(12)^ and WHO^(11)^ in 89 % and 57 % of participants, respectively (data not shown). FS were dose-dependently and significantly related to all-cause mortality (Fig. 1(a)). The nadir was observed at 25 g/d FS and mean (CI) HR increased to 1·01, 95 % CI (0·99, 1·03) and 1·12, 95 % CI (1·08, 1·17) at 50 g/d and 100 g/d FS, respectively, as compared with 25 g/d FS (Fig. 1(a)). In contrast, intrinsic sugars were not significantly associated with all-cause mortality (Fig. 1(b)). FS remained dose-dependently related to mortality in sensitivity analyses removing the following participants: death within the first 2 years of follow-up (landmark analysis; online Supplementary Fig. 4(a)), unintentional weight loss (online Supplementary Fig. 5(a)), history of CVD and cancer (online Supplementary Fig. 6(a)), non-typical diet (online Supplementary Fig. 7(a)), Oxford WebQ completed for a weekend day (online Supplementary Fig. 8(a)), only one (online Supplementary Fig. 9(a)) or up to two (online Supplementary Fig. 10(a)) completed Oxford WebQ. The association between FS and mortality was still significant if only the first Oxford WebQ was considered (online Supplementary Fig. 11(a)). FS remained significantly and dose-dependently associated with mortality if WHR and height were included as covariates instead of BMI (online Supplementary Fig. 12(a)), if models were further adjusted for the diet quality score (online Supplementary Fig. 13(a)), or if history of psychiatric disease was removed as a covariate (online Supplementary Fig. 14(a)). Similar to the primary analyses, intrinsic sugars were not significantly associated with all-cause mortality in all sensitivity analyses (online Supplementary Fig. 4(b)–14(b)) except after removal of participants with up to two completed Oxford WebQ (online Supplementary Fig. 10(b)).

**Table 2.** Dietary intake of the UK Biobank cohort*

Parameters			Quintiles of FS intake (g/d)									
	Total cohort (*n* 186 811)		0·0 to 32·7 (*n* 37 362)		32·7 to 48·8 (*n* 37 362)		48·8 to 65·4 (*n* 37 362)		65·4 to 88·6 (*n* 37 362)		88·6 to 461·4 (*n* 37 363)	
	Mean	SD	Mean	SD	Mean	SD	Mean	SD	Mean	SD	Mean	SD
Carbohydrates	259·3	73·9	200·0	55·6	229·0	52·1	252·2	52·2	279·4	54·3	335·9	71·2
Total sugars	129·9	49·0	85·5	31·3	107·0	28·9	123·7	29·0	143·7	30·0	189·6	46·2
Intrinsic sugars	66·9	29·8	64·6	29·9	66·0	28·5	66·9	28·5	67·8	29·2	69·3	32·5
FS	63·0	37·4	20·9	8·5	41·0	4·6	56·9	4·8	75·9	6·6	120·3	32·9
FS beverages	26·8	28·4	5·4	7·1	13·6	10·9	21·3	13·8	31·6	17·6	62·2	37·8
Soda/fruit drinks	10·1	21·4	0·7	2·9	2·7	6·7	5·3	9·9	10·3	14·9	31·5	36·1
Juice	11·0	14·5	2·8	5·9	7·5	9·3	11·1	11·6	14·4	13·8	19·4	21·0
Milk-based drinks	1·8	5·1	0·6	2·4	1·2	3·6	1·7	4·4	2·3	5·5	3·4	7·6
Tea/coffee	3·3	8·6	0·8	3·0	1·7	4·9	2·7	6·7	4·0	8·9	7·4	13·5
FS solids	36·2	22·3	15·5	8·6	27·4	11·0	35·6	13·7	44·3	17·4	58·2	27·4
Treats	24·3	18·9	9·6	7·6	17·7	10·4	23·3	12·7	29·7	15·8	41·2	25·1
Cereals	2·6	3·8	1·7	3·0	2·3	3·4	2·6	3·7	2·9	4·0	3·4	4·5
Toppings	6·4	8·7	2·0	4·5	4·7	6·9	6·7	8·2	8·4	9·3	10·0	10·7
Sauces	1·4	2·1	1·0	1·8	1·3	1·9	1·5	2·0	1·6	2·2	1·8	2·5
Alcohol	17·3	22·1	21·8	25·8	18·4	22·1	17·0	20·9	15·6	20·0	13·6	20·2
Fat	79·3	28·0	64·9	23·2	72·1	23·2	78·5	24·3	85·0	26·3	95·9	31·6
Protein	74·8	20·8	69·6	20·3	71·5	19·0	74·1	19·3	76·7	19·9	81·7	22·9
Fibre	19·0	7·0	17·9	7·1	18·6	6·7	19·0	6·7	19·5	6·9	20·2	7·6
Energy (kJ/d)	9115	2319	7618	1862	8311	1822	8946	1867	9653	1974	11 045	2409

FS, free sugar.

* All nutrients are presented as mean (sd) and in g/d.

### Free sugars in beverages *v*. free sugars in solids and all-cause mortality

Mean (sd) consumption of FS in beverages and FS in solids was 26·8 (28·4) and 36·2 (22·3) g/d, respectively (Table 2). FS in beverages were dose-dependently and significantly associated with all-cause mortality (Fig. 1(c)). The nadir was observed at 0 g/d FS and mean (CI) HR increased to 1·10, 95 % CI (1·07, 1·14) and 1·28, 95 % CI (1·19, 1·39) at 50 g/d and 100 g/d FS, respectively (Fig. 1(c)). The association between FS in beverages and mortality remained similar in all sensitivity analyses (online Supplementary Fig. 4(c)–14(c)). FS in solids were not significantly associated with all-cause mortality in the primary (Fig. 1(d)) and in all sensitivity analyses (online Supplementary Fig. 4(d) to 14(d)) except after removal of participants with a history of CVD and cancer (online Supplementary Fig. 6(d)).

### Free sugars in beverage subtypes and all-cause mortality

**Fig. 2.**
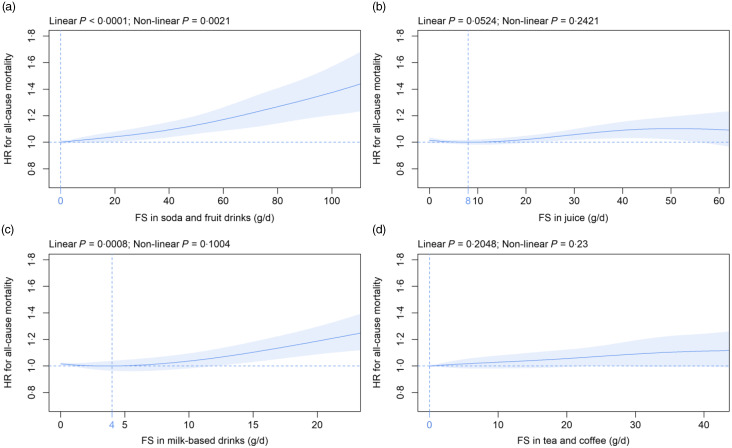
Association of free sugars (FS) in (a) soda/fruit drinks, (b) juice, (c) milk-based drinks and (d) tea/coffee (all g/d) with all-cause mortality. Models are adjusted and presented as indicated in Fig. 1.

Mean (sd) consumption of FS in beverage subtypes was as follows: soda/fruit drinks 10·1 (21·4), juice 11·0 (14·5), milk-based drinks 1·8 (5·1) and tea/coffee 3·3 (8·6) g/d (Table 2). FS in soda/fruit drinks were dose-dependently and significantly associated with all-cause mortality with the nadir observed at 0 g/d and mean (CI) HR increased to 1·04, 95 % CI (1·00, 1·08) and 1·09, 95 % CI (1·04, 1·15) at 20 g/d and 40 g/d FS, respectively (Fig. 2(a)), with a standard serving containing 33 g FS. FS in milk-based drinks were dose-dependently and significantly related to all-cause mortality with the nadir detected at 4 g/d and mean (CI) HR increased to 1·19, 95 % CI (1·09, 1·30) at 20 g/d as compared with 4 g/d (Fig. 2(c)), with a standard serving containing 16 g FS. In contrast, FS in juice (Fig. 2(b)) and tea/coffee (Fig. 2(d)) were not significantly associated with all-cause mortality. These findings were robust in all sensitivity analyses with the following exceptions: The association with mortality was significant for juice if participants with unintentional weight loss were removed (online Supplementary Fig. 5(f)) and after adjustment for the diet quality score (online Supplementary Fig. 13(f)). The relation between FS in milk-based drinks and mortality did not remain statistically significant if only the first Oxford WebQ was considered (online Supplementary Fig. 11(g)). FS in tea/coffee were significantly associated with mortality if participants with unintentional weight loss (online Supplementary Fig. 5(h)) or with up to two completed Oxford WebQ (online Supplementary Fig. 10(h)) were removed.

### Free sugars in solids subtypes and all-cause mortality

**Fig. 3.**
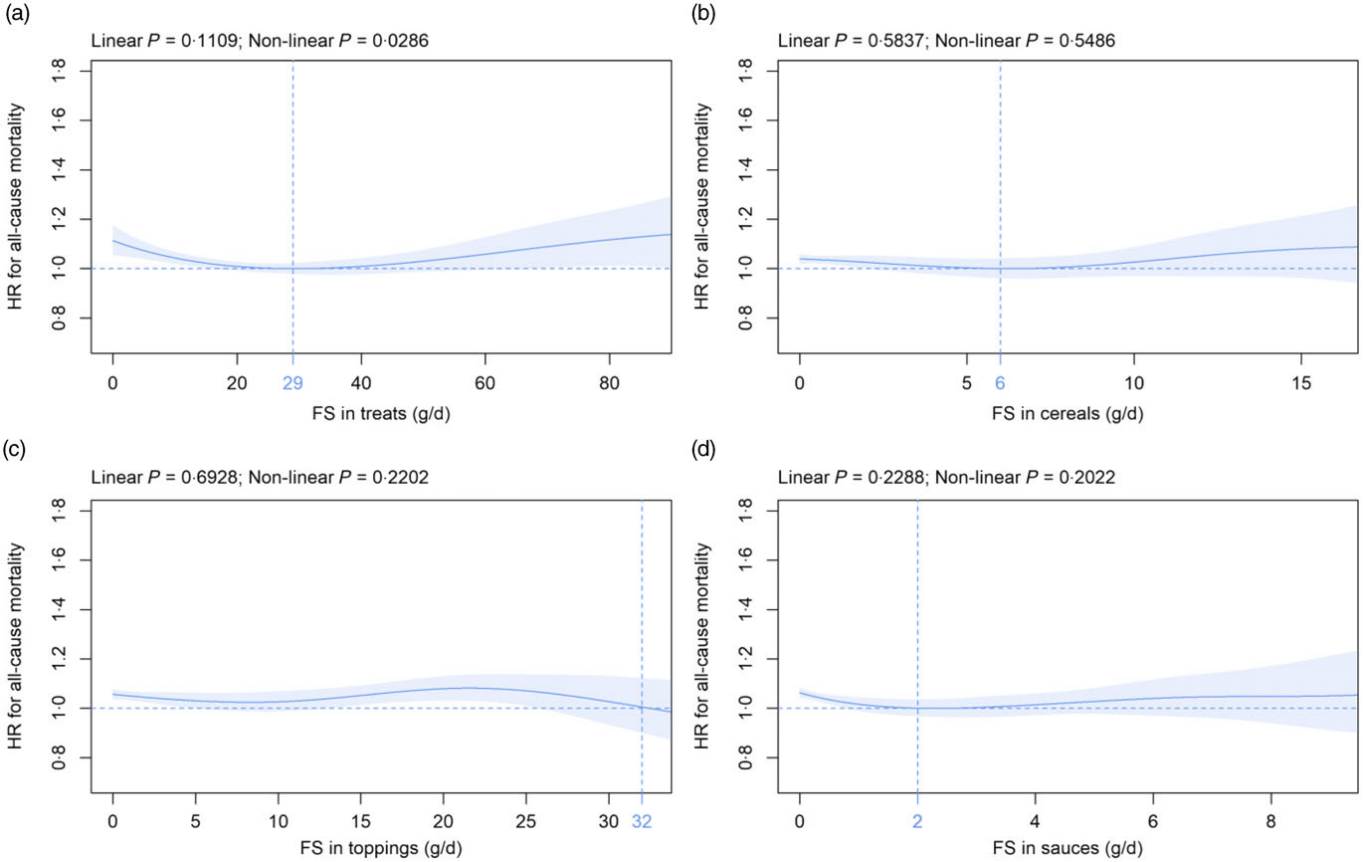
Association of free sugars (FS) in (a) treats, (b) cereals, (c) toppings and (d) sauces (all g/d) with all-cause mortality. Models are adjusted and presented as indicated in Fig. 1.

Mean (sd) consumption of FS in solids subtypes was as follows: treats 24·3 (18·9), cereals 2·6 (3·8), toppings 6·4 (8·7) and sauces 1·4 (2·1) g/d (Table 2). Within solids subtypes, FS in treats were significantly associated with mortality in a non-linear fashion in the primary (Fig. 3(a)) and in five (online Supplementary Fig. 6i, 8i, 12i to 14i) of the eleven sensitivity analyses. In contrast, the FS in cereals, toppings and sauces were not significantly associated with all-cause mortality over the whole range of consumption in all analyses (Fig. 3(b), (c), (d) and online Supplementary Fig. 4(j), (k), (l) to 14(j), (k), (l)) except for FS in sauces if participants with at least one Oxford WebQ completed for a weekend day were removed (online Supplementary Fig. 8(l)).

## Discussion

### Principal findings

To the best of our knowledge, this is the first study that systematically assesses the association of FS from all relevant sources with all-cause mortality and some sugar sources are studied for the first time. Non-linear associations between FS subtypes and mortality are defined, whereas previous research has commonly focused on linear relations.

A significant dose-dependent association exists between FS consumption and all-cause mortality with mean FS intake of the UK Biobank population being beyond consumption levels recommended by the WHO^(11)^ and the NHS England^(12)^. In contrast, intrinsic sugars are not significantly related to all-cause mortality over the whole range of consumption although mean intake of FS and intrinsic sugars is comparable. FS in beverages are significantly associated with all-cause mortality. Within this category, FS in soda/fruit drinks and milk-based drinks are dose-dependently related to mortality, whereas no significant association is found for juice and tea/coffee. In contrast to beverages, FS in solids are not significantly linked to all-cause mortality and only for FS in treats a significant non-linear association is observed. All results are largely consistent in various sensitivity analyses except for FS in treats for which only five out of eleven sensitivity analyses show a significant relation with mortality. Our results highlight that associations between FS and all-cause mortality depend on FS source.

### Comparison with other studies

FS intake of UK Biobank participants in the present analysis (median: 56·8 g/d) is higher as compared with representative data for the UK population from the National Diet and Nutrition Survey (median: 44·8 g/d)^(37)^. These findings support recent analyses that the UK Biobank cohort is not demographically representative of the general UK population^(38)^. However, differences in age distribution, i.e. 19 to 64 years in the study by Amoutzopoulos *et al*.^(37)^ as compared with 39 to 72 years in the present analysis might also contribute.

FS consumption and all-cause mortality are significantly and dose-dependently associated in our study with the nadir observed at 25 g/d. Few studies have assessed this association, and these have reported conflicting results^(13,14,39)^. In accordance with the current findings, the fourth as compared with the first quartile of FS consumption is associated with a significantly increased risk for all-cause mortality in both women and men in a study from Japan^(14)^. In contrast, a U-shaped association between FS intake and all-cause mortality is demonstrated in another study from Sweden with lowest risk of death observed in the 7·5 to <10 % energy from FS intake category^(13)^. Yet another study from the USA does not find any significant link between FS consumption and all-cause mortality in both sexes^(39)^. Different proportions of FS subtypes in the respective studies might contribute to the conflicting findings.

To the best of our knowledge, the current study is the first to analyse the association of intrinsic sugars with all-cause mortality. In contrast to FS, intrinsic sugars are not significantly related to all-cause mortality. The majority of intrinsic sugars is incorporated within the structure of intact fruit and vegetables or is naturally present as lactose and galactose in milk^(11)^. The present results support the recommendation by the WHO that intrinsic sugars and FS need to be distinguished since their physiological impact differs with FS but not intrinsic sugars showing adverse effects^(11)^. Published studies have focused on food groups rich in intrinsic sugars, e.g. fruits, vegetables and dairy products, but not on the sugars they naturally contain. A dose-dependent negative association exists between fruit and vegetable consumption and all-cause mortality in a meta-analysis comprising ninety-five prospective studies^(40)^. A lower risk is seen for fruit and vegetable intake of up to 800 g/d with a 31 % reduction as compared with non-consumption^(40)^. For dairy products, no significant association with all-cause mortality is found in a meta-analysis of twenty-nine prospective studies^(41)^. It remains to be elucidated whether intrinsic sugars per se are neutral concerning mortality or whether adverse effects of this sugar subtype are neutralised by other beneficial ingredients and/or the plant matrix found in intrinsic sugar-rich sources^(42)^.

FS in beverages are dose-dependently associated with all-cause mortality in our report. Within this category, FS in soda/fruit drinks are related to mortality. Most published studies have assessed the association between sugar-sweetened beverage servings and mortality. Intake is significantly related to all-cause mortality in most^(13,36,43–46)^ but not all^(47,48)^ studies. The definition of sugar-sweetened beverages is heterogeneous with juice being included^(13)^, excluded^(43,44,46,48)^ or analysed separately^(36,45,47)^ in different studies. This is of importance since FS in juice are not significantly associated with all-cause mortality in the present analysis, although mean FS intake from soda/fruit drinks and juice is comparable. The current results regarding FS in soda/fruit drinks *v*. juice are in accordance with recent data from Anderson and co-workers who also assessed UK Biobank data using a different approach^(36)^. The authors demonstrate convincingly that HR for mortality is increased for sugar-sweetened beverages but not for juice if >2 servings/d are compared with non-consumption^(36)^. To the best of our knowledge, the current study is the first to suggest a positive link between FS in milk-based drinks and all-cause mortality. These results indicate that sugary milk drinks might have adverse effects on mortality similar to the well-established impact of soda/fruit drinks. It is important to note in this context that milk-based drinks are not liable for the Soft Drinks Industry Levy in the UK, i.e. the ‘sugar tax’^(49)^ despite showing a similar association with all-cause mortality in the present study as compared with FS from soda/fruit drinks. It remains to be elucidated why FS in tea/coffee are not significantly associated with mortality in contrast to FS in soda/fruit drinks and milk-based drinks. It is interesting to note in this context that our group has recently demonstrated that tea and to a lesser extent coffee consumption are negatively related to all-cause mortality in UK Biobank participants^(32)^. Therefore, it is well possible that adverse effects of FS concerning mortality are somewhat blunted by positive health effects of tea/coffee intake. Alternatively or in addition, there might be some residual confounding despite adjustment for multiple covariates and various sensitivity analyses performed in the current study, i.e. tea/coffee drinkers might be different from consumers of soda/fruit drinks and milk-based drinks. Furthermore, it needs to be pointed out that mean intake of FS from tea/coffee is much lower in UK Biobank participants as compared with FS from soda/fruit drinks and juice. The effect of adding sugar to tea/coffee has been a somewhat neglected research subject so far despite its relevance in everyday life. Thus, no prospective study exists defining the association between sugars in tea/coffee and mortality, as well as morbidity. Three independent studies using a cross-sectional design have yielded conflicting results with sugar added to tea/coffee being positively^(50)^, negatively^(51)^ or not^(52)^ linked to adverse metabolic parameters.

While FS in beverages are positively associated with HR for all-cause mortality, we observe no link for FS in solids. To the best of our knowledge, only one study to date has analysed the relation between FS in solids and mortality risk^(39)^. Using a different approach, the highest as compared with the lowest quintile of added sugars in solids intake is associated with a significantly decreased mortality risk in both women and men^(39)^. Combined, the current study and the results by Tasevska and co-workers^(39)^ suggest that FS in solids are not linked to an increased all-cause mortality risk. Within solids subtypes, FS in treats are associated with all-cause mortality in a non-linear fashion, and the nadir is observed at 29 g/d in the current analysis. However, these results remain statistically significant in only about half of the sensitivity analyses. It is interesting to note in this context that servings of treats are significantly and inversely related to all-cause mortality in an independent report^(13)^. The authors speculate that consumption of treats is positively linked to social interactions, e.g. breaks at work with coffee and pastries^(13)^. Therefore, lower intake might be related to fewer social connections, which, in turn, is associated with higher mortality^(13)^. All other FS in solids subtypes are not significantly related to mortality in the present analysis. For toppings, a trend toward an inverse relation has been demonstrated recently^(13)^. To the best of our knowledge, no study so far has assessed the association between FS in cereals and sauces with all-cause mortality.

Together, these data suggest that FS from beverages and solids might show distinct physiological effects and that FS from solids are not linked with all-cause mortality. It is interesting to note in this context that significant differences concerning subjective feelings of hunger, fullness and satiety can be observed between liquid and solid carbohydrate foods despite similar effects on glycaemic and insulin responses^(53)^.

Several potential mechanisms by which FS increase mortality exist. Thus, high FS intake might cause metabolic and CVD via induction of oxidative stress^(54)^ and proinflammatory cytokines like tumor necrosis factor *α* ^(55)^, as well as by displacing nutritionally superior foods, stimulation of insulin resistance and damaging the intestine with concomitantly decreased nutrient absorption^(56)^.

### Strengths and limitations of this study

Strengths of the current study include a large sample size, the prospective cohort design, thorough characterisation of participants, mean follow-up >12 years, a wide range of sugar subtype intake, as well as analyses with penalised cubic splines to allow non-linear predictor effects. Limitations include residual confounding, as well as measurement errors in the assessment of the exposure variables and potential confounders. All consumption data have not been independently assessed but self-reported. In addition, about 38 % and 61 % of participants have completed only one and up to two Oxford WebQ, respectively, which might limit representativity of data^(24)^. However, all major findings concerning the association of FS subtype consumption with mortality are similar in sensitivity analyses in participants with at least two and three Oxford WebQ questionnaires filled out, respectively. Furthermore, the intake of FS from other solid foods than treats, i.e. cereals, toppings and sauces, is rather low and might contribute to the non-significant results observed. In addition, participants could choose ‘varied’ for sugar added to tea/coffee, and ‘varied’ was set to one teaspoon in the present analysis since this was the portion size most commonly chosen by all participants. Furthermore, standard portion sizes are used in the current study, but the real portion sizes consumed might vary between individuals especially for solid foods. Moreover, a ‘healthy volunteer’ selection bias is possible since the cohort is not demographically representative of the general UK population^(38)^. However, a representative population is not required to define exposure–disease relationships^(38)^.

### Conclusions

FS but not intrinsic sugars are associated with all-cause mortality. Furthermore, FS in beverages are significantly and dose dependently related to mortality, whereas no association is found for FS in solids. Within beverages, a significant dose-dependent association with mortality is detected for FS in soda/fruit drinks and milk-based drinks, while no significant association is found for FS in juice and tea/coffee. Interventions targeting FS subtypes might be most effective concerning mortality if focused on the reduction of soda/fruit drinks and milk-based sugary drinks; however, the present results need to be confirmed by independent studies. Further prospective studies on sugar subtype intake in relation to morbidity from metabolic and CVD, as well as cancer, are necessary to provide even more definitive conclusions.

## Supplementary material

For supplementary material accompanying this paper visit https://doi.org/10.1017/S0007114522003233. click here to view supplementary material
