# Supplementary material for: Association of all-cause mortality with sugar intake from different sources in the prospective cohort of UK Biobank participants
Source: Br J Nutr. 2022 Oct 7;130(2):294–303. doi: 10.1017/S0007114522003233 (PMC10277665; doi:10.1017/S0007114522003233)
Supplement: Supplementary file 1 [file S0007114522003233sup001.docx]

**Supplementary Material**

## Association of all-cause mortality with sugar intake from different sources in the prospective cohort of UK Biobank participants

Anna Kaiser1#*; Sylva M Schaefer1#; Inken Behrendt1; Gerrit Eichner2§; Mathias Fasshauer1,3§

1. Institute of Nutritional Science, Justus-Liebig University of Giessen, Giessen, Germany.
2. Mathematical Institute, Justus-Liebig University of Giessen, Giessen, Germany.
3. Department of Internal Medicine (Endocrinology, Nephrology, and Rheumatology), University of Leipzig, Leipzig, Germany.

#AK and SMS contributed equally to this work and are joint first authors.

§GE and MF contributed equally to this work and are joint senior authors.

*Corresponding author. Mailing address: Goethestr. 55, 35390 Giessen, Germany. Phone:

+49 641 9939067. E-mail: [anna.kaiser@ernaehrung.uni-giessen.de](mailto:anna.kaiser@ernaehrung.uni-giessen.de)

## Supplementary Table 1

Six-step procedure to estimate FS content

## Supplementary Figure 1

Sugar sources relevant to the present study

Free sugars (FS) are all monosaccharides and disaccharides added to foods by the manufacturer, cook, or consumer, plus sugars naturally present in honey, syrups, and fruit juices.

FS were divided into FS in beverages and FS in solids. The following sugar- containing beverage subtypes were defined: soda/fruit drinks (i.e., carbonated drinks, fruit drinks, J20, squash, cordial, excluding low calorie or diet drinks), pure juice (i.e., fruit and vegetable; indicated as “juice” throughout the manuscript), milk-based drinks (i.e., dairy/yogurt-based smoothies, yogurt drinks, flavoured milk or milkshakes, hot chocolate or other milk-based drinks, excluding plain milk), and sugar added to tea/coffee. For tea/coffee, participants could choose the amount of added sugar per drink as half, one, two, or three teaspoon(s), as well as “varied”. This number of teaspoons was multiplied by the total number of cups of tea and coffee consumed, respectively. Since one teaspoon was the most commonly chosen portion size for added sugar in tea/coffee, this amount was set if participants indicated “varied”. The following sugar-containing solids subtypes were defined: treats (i.e., pastries, candies, chocolate, ice cream, sweetened yoghurt), breakfast cereals (i.e., all food items labelled as “cereal(s)”, porridge, muesli, shreddies; indicated as “cereals” throughout the manuscript), toppings (i.e., table sugar, jam, honey, syrup, peanut butter, chocolate/nut spread, stewed/cooked fruit), and sauces (i.e., all food items labelled as “sauce(s)”, “salad cream”, mayonnaise, ketchup, chutney, salad dressing, pesto, gravy).

## Supplementary Figure 2

Flowchart of participant selection

## Supplementary Figure 3

Venn diagram depicting number of participants excluded by six exclusion criteria

## Supplementary Figure 4

Landmark analysis

Association of (a) FS, (b) intrinsic sugars, as well as FS in (c) beverages, (d) solids,

(e) soda/fruit drinks, (f) juice, (g) milk-based drinks, (h) tea/coffee, (i) treats, (j) cereals, (k) toppings, and (l) sauces (all g/d) with all-cause mortality (landmark analysis; n=186,529; number of deaths=8337). Models are adjusted for energy intake, age, sex, ethnic background, BMI, SBP, Townsend deprivation index, general health status, total household income, highest qualification, smoking status, alcohol intake, physical activity, and history of psychiatric disease. Covariates not fulfilling the proportional hazard assumption are stratified. The nadir is indicated in blue. FS, Free sugars; HR, Hazard ratio.

## Supplementary Figure 5

Unintentional weight loss removed

Association of (a) FS, (b) intrinsic sugars, as well as FS in (c) beverages, (d) solids,

(e) soda/fruit drinks, (f) juice, (g) milk-based drinks, (h) tea/coffee, (i) treats, (j) cereals, (k) toppings, and (l) sauces (all g/d) with all-cause mortality (unintentional weight loss removed; n=157,213; number of deaths=7073). Models are adjusted and presented as indicated in Supplementary Fig. 4. FS, Free sugars; HR, Hazard ratio.

## Supplementary Figure 6

Participants with history of cardiovascular disease and cancer removed

Association of (a) FS, (b) intrinsic sugars, as well as FS in (c) beverages, (d) solids,

(e) soda/fruit drinks, (f) juice, (g) milk-based drinks, (h) tea/coffee, (i) treats, (j) cereals, (k) toppings, and (l) sauces (all g/d) with all-cause mortality (Participants with history of cardiovascular disease and cancer removed; n=165,003; number of deaths=6325). Models are adjusted and presented as indicated in Supplementary Fig. 4. FS, Free sugars; HR, Hazard ratio.

## Supplementary Figure 7

Non-typical diet removed

Association of (a) FS, (b) intrinsic sugars, as well as FS in (c) beverages, (d) solids,

(e) soda/fruit drinks, (f) juice, (g) milk-based drinks, (h) tea/coffee, (i) treats, (j) cereals, (k) toppings, and (l) sauces (all g/d) with all-cause mortality (non-typical diet

removed; n=125,435; number of deaths=6246). Models are adjusted and presented as indicated in Supplementary Fig. 4. FS, Free sugars; HR, Hazard ratio.

## Supplementary Figure 8

Participants with at least one Oxford WebQ completed for a weekend day removed Association of (a) FS, (b) intrinsic sugars, as well as FS in (c) beverages, (d) solids,

(e) soda/fruit drinks, (f) juice, (g) milk-based drinks, (h) tea/coffee, (i) treats, (j) cereals, (k) toppings, and (l) sauces (all g/d) with all-cause mortality (Participants with at least one Oxford WebQ completed for a weekend day removed; n=95652; number of deaths=4684). Models are adjusted and presented as indicated in Supplementary Fig. 4. FS, Free sugars; HR, Hazard ratio.

## Supplementary Figure 9

Participants with one completed Oxford WebQ removed

Association of (a) FS, (b) intrinsic sugars, as well as FS in (c) beverages, (d) solids,

(e) soda/fruit drinks, (f) juice, (g) milk-based drinks, (h) tea/coffee, (i) treats, (j) cereals, (k) toppings, and (l) sauces (all g/d) with all-cause mortality (Participants with one completed Oxford WebQ removed; n=115,595; number of deaths=4867). Models are adjusted and presented as indicated in Supplementary Fig. 4. FS, Free sugars; HR, Hazard ratio.

## Supplementary Figure 10

Participants with up to two completed Oxford WebQ removed

Association of (a) FS, (b) intrinsic sugars, as well as FS in (c) beverages, (d) solids,

(e) soda/fruit drinks, (f) juice, (g) milk-based drinks, (h) tea/coffee, (i) treats, (j) cereals, (k) toppings, and (l) sauces (all g/d) with all-cause mortality (Participants with up to two completed Oxford WebQ removed; n=72337; number of deaths=2961). Models are adjusted and presented as indicated in Supplementary Fig. 4. FS, Free sugars; HR, Hazard ratio.

## Supplementary Figure 11

First Oxford WebQ only

Association of (a) FS, (b) intrinsic sugars, as well as FS in (c) beverages, (d) solids,

(e) soda/fruit drinks, (f) juice, (g) milk-based drinks, (h) tea/coffee, (i) treats, (j)

cereals, (k) toppings, and (l) sauces (all g/d) with all-cause mortality. Only the first Oxford WebQ was used for intake estimation (n=186,811; number of deaths=8576). Models are adjusted and presented as indicated in Supplementary Fig. 4. FS, Free sugars; HR, Hazard ratio.

## Supplementary Figure 12

Adjustment for WHR and height instead of BMI

Association of (a) FS, (b) intrinsic sugars, as well as FS in (c) beverages, (d) solids,

(e) soda/fruit drinks, (f) juice, (g) milk-based drinks, (h) tea/coffee, (i) treats, (j) cereals, (k) toppings, and (l) sauces (all g/d) with all-cause mortality. Models were adjusted for WHR and height instead of BMI (n=186,769; number of deaths=8569). Models are adjusted and presented as indicated in Supplementary Fig. 4. FS, Free sugars; HR, Hazard ratio.

## Supplementary Figure 13

Adjustment for diet quality score

Association of (a) FS, (b) intrinsic sugars, as well as FS in (c) beverages, (d) solids,

(e) soda/fruit drinks, (f) juice, (g) milk-based drinks, (h) tea/coffee, (i) treats, (j) cereals, (k) toppings, and (l) sauces (all g/d) with all-cause mortality. Models were further adjusted for diet quality score (n=184,455; number of deaths=8424). Models are adjusted and presented as indicated in Supplementary Fig. 4. FS, Free sugars; HR, Hazard ratio.

## Supplementary Figure 14

Not adjusted for psychiatric disease

Association of (a) FS, (b) intrinsic sugars, as well as FS in (c) beverages, (d) solids,

(e) soda/fruit drinks, (f) juice, (g) milk-based drinks, (h) tea/coffee, (i) treats, (j) cereals, (k) toppings, and (l) sauces (all g/d) with all-cause mortality. Models were not adjusted for psychiatric disease (n=186,811; number of deaths=8576). Models are adjusted and presented as indicated in Supplementary Fig. 4. FS, Free sugars; HR, Hazard ratio.

**Supplementary Table 1** Six-step procedure to estimate Free sugars (FS) content.1

| **Step 1** | Food items containing 0 g of total sugars are assigned 0 g of FS. |
| --- | --- |
| **Step 2** | Food items in the food groups stated below are unprocessed or minimally processed with no FS and are assigned 0 g of FS: |
|  | 1. spices and herbs 2. fats and oils 3. plain cereal grains, pseudocereals (e.g., buckwheat, quinoa, amaranth),   ﬂour, pasta, rice, plain cereal products, unsweetened potato chips   1. plain breads with minimal amounts of FS only used for activation of yeast in fermentation (<9 g/>1000 g pre-baking weight) 2. eggs and egg products (excluding egg-based desserts) 3. fresh, frozen, or cooked fruits, berries, vegetables (including salads without dressing and root vegetables), unsweetened dried fruits 4. fruit and vegetables canned in 100% vegetable juice or in artiﬁcially   sweetened liquid   1. unsweetened nuts, seeds, coconut, coconut products 2. fresh meat, fresh ﬁsh, fresh seafood, tofu, unsweetened legumes,   mushrooms, mixed meat dishes without FS   1. coﬀee, tea, alcoholic beverages unsweetened or artiﬁcially sweetened 2. unsweetened milk, unsweetened dairy products, non-dairy milk substitutes (e.g., oat and soy drinks and yoghurt) 3. 100% vegetable juices; vegetable drinks sweetened with artiﬁcial   sweeteners only   1. jams, beverage bases, fruit curds or sauces that are unsweetened or artificially sweetened |

| **Step 3** | Food items in the food groups stated below are considered having minimal amounts of intrinsic sugars and are assigned 100% of total sugars as FS (total sugar values were estimated based on McCance and Widdowson’s The Composition of Foods (1) and its supplements (2–10)): |
| --- | --- |
|  | 1. confectionary except confectionary with dairy (e.g., chocolate, fudge) 2. ﬂavoured potato chips and other salty snacks 3. breakfast cereals and bars (e.g., muesli bars) without fruits, chocolate, or dairy 4. sugar-sweetened coﬀee and tea, beverage and soup bases with FS and   without dairy (e.g., cordial)   1. processed meats, ﬁsh, shellﬁsh, blood products, and vegetarian dishes   including pies, ﬁlled pastries, and breaded meats   1. sugar-sweetened soda, sports drinks, ﬂavoured water, and energy   drinks without fruits   1. baked food items such as cookies, buns, donuts, sponge cake, and other batter-based products without fruits, chocolate, or dairy 2. plain pastries without ﬁllings, fruits, dairy, nuts, or chocolate 3. sugar-sweetened breads without fruits or dairy 4. sugar-sweetened non-dairy milk substitutes (e.g., oat and soy drinks and yoghurt) without fruits 5. table sugar, honey, and syrups 6. sugar-sweetened non-dairy alcoholic beverages (e.g., punch) 7. food items with FS that are pickled, cured, or marinated (e.g., pickled vegetables) 8. sauces, dressings, and mayonnaise that contain FS 9. fruit juice, fruit juice concentrate |
| **Step 4** | For food items not covered by steps 1 to 3, with analytical information on lactose available from (1–10), and not containing any fruits or vegetables, FS are calculated as sum of total sugars minus lactose. |

| **Step 5** | For composite food items not already covered by steps 1 to 4 and with total sugars content available from (1–10), FS content is calculated as follows: |
| --- | --- |
|  | 1. Recipes are taken from the UKDA recipe database (11) or from McCance and Widdowson (1–10) 2. FS and total sugars for individual recipe items are defined by steps 1 to 4 3. The amount of FS and total sugars of each individual recipe item is multiplied with the percentage of this item within the recipe to calculate its content in 100 g recipe 4. The individual recipe items are summed up to calculate FS and total sugar content in the composite food item 5. The proportion of FS to total sugars is determined 6. This proportion is multiplied with total sugars content from (1–10) |
| **Step 6** | For composite food items not already covered by steps 1 to 5 and with total sugars content **not** available from (1–10), FS content is calculated as follows: |
|  | 1. Recipes are taken from the UKDA recipe database (11) or from McCance and Widdowson (1–10) 2. FS for individual recipe items are defined by steps 1 to 4 3. The amount of FS of each individual recipe item is multiplied with the percentage of this item within the recipe to calculate its content in 100 g recipe 4. The individual recipe items are summed up to calculate FS content in   the composite food item |

1FS are all monosaccharides and disaccharides added to foods by the manufacturer, cook, or consumer, plus sugars naturally present in honey, syrups, and fruit juices.

## References

1. McCance RA & Widdowson EM (2002) *McCance and Widdowson's: The Composition of Foods: sixth summary edition,* 06th edn. Cambridge: Royal Society of Chemistry.
2. Holland B, Welch A, Buss D (1992, repr. 1996) *Vegetable dishes: Second Supplement to McCance and Widdowson's the composition of Foods, 5th ed.* Cambridge: Royal Society of Chemistry.
3. Holland B, Unwin I, Buss D (1992) *Fruit and Nuts: First Supplement to the Fifth Edition of McCance and Widdowson’s The Composition of Foods, 5th ed.* Cambridge: RSC Publishing.
4. Holland B, Unwin I d., Buss DH *et al.* (1989) *Milk Products and Eggs: Fourth Supplement to McCance and Widdowson's the Composition of Foods, 4th ed.,* 4th edn. Supplement / Robert Alexander MacCance; Elsie M. Widdowson Hrsg. The Royal

Society of Chemistry; 4: *The composition of foods*. Cambridge, UK, London: Royal Society of Chemistry; Ministry of Agriculture Fisheries and Food.

1. Holland B, Unwin I, Buss D (editors) (1991) *Vegetable, Herbs and Spices: Fifth Supplement to McCance and Widdowson's the Composition of Foods, 5th ed.* Cambridge, UK, London: Royal Society of Chemistry; Ministry of Agriculture Fisheries and Food.
2. Holland B, Brown J, Buss DH *et al.* (editors) (1993) *Fish and Fish products: Third supplement to McCance and Widdowson’s the Composition of Food, 5th ed.* Cambridge, UK, London: Royal Society of Chemistry; Ministry of Agriculture Fisheries and Food.
3. Chan W, Bron J, Church S *et al.* (1996) *Meat products and Dishes: Sixth Supplement to McCance and Widdowson's The composition of Foods, 5th ed.* Cambridge: Royal Society of Chemistry and Ministry of Agriculture Fisheries and Food.
4. Chan W, Brown J, Lee S *et al.* (1995) *Meat poultry and Game: Fifth supplement to McCance and Widdowson's The composition of Foods, 5th ed.* Cambridge, London: Royal Society of Chemistry; Ministry of Agriculture Fisheries and Food.
5. Chan W, Buss DH, Brown J (1994) *Miscellaneous Foods: Fourth supplement to McCance and Widdowson's the composition of Foods, 5th ed.* Cambridge: Royal Society of Chemistry.
6. McCance RA & Widdowson EM (2014) *McCance and Widdowson's: The Composition of Foods: seventh summary edition*. Royal Society of Chemistry.
7. MRC Human Nutrition Research (2017) Food Standards Agency Standard Recipes Database, 1992-2012. [data collection]. *UK Data Service. SN: 8159*.

FS in soda/fruit drinks

FS in juice

FS in beverages

FS in milk-based

drinks

FS in tea/coffee

Free sugars (FS)

Total sugars FS in treats

Intrinsic sugars

FS in cereals

FS in solids

FS in toppings

FS in sauces

287,655 Excluded because Oxford WebQ not filled out

498,618 participants

24,152 Excluded because

fulfilling at least one exclusion criterion

(Fig. S3 for further details)

210,963 participants

3794 Excluded because of

removing pilot data

502,412 participants at

baseline

186,811 participants included in analysis


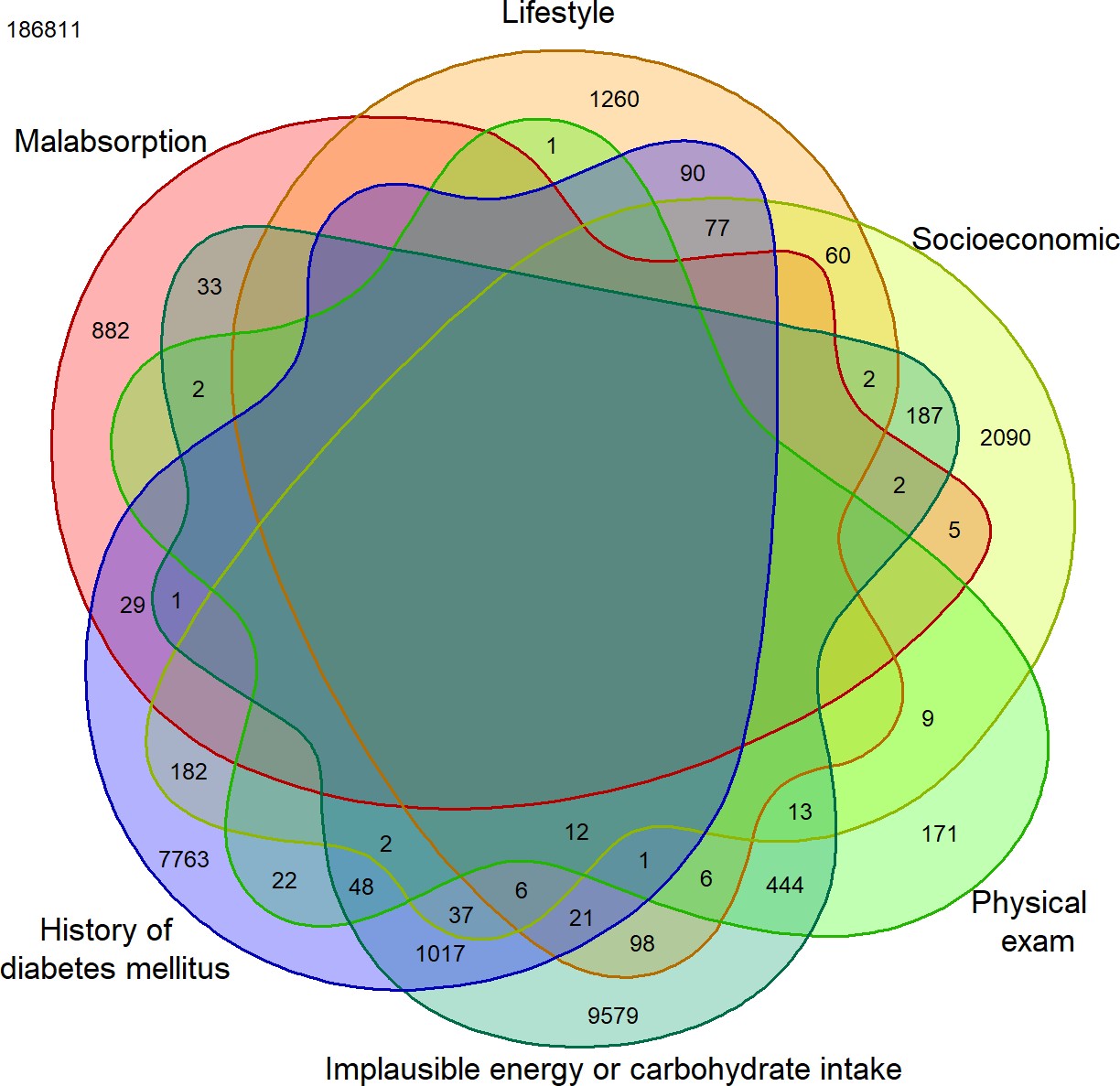


# (a) (b)


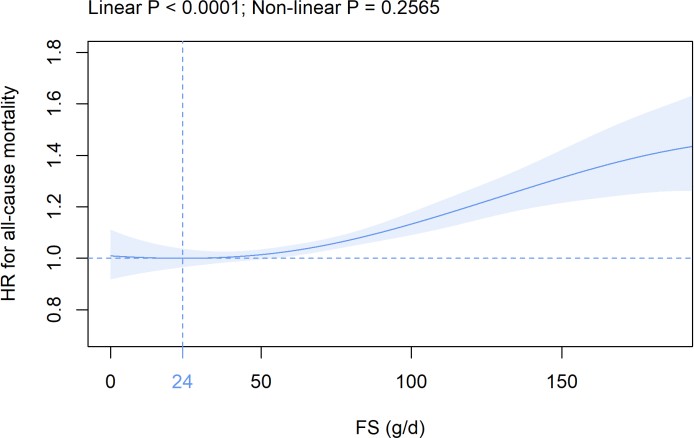

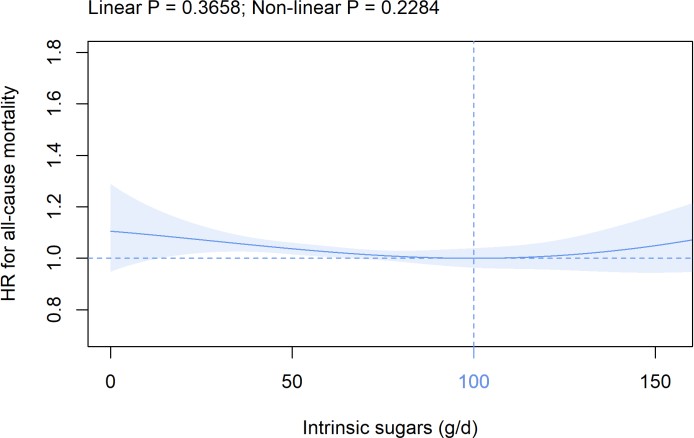


(c) (d)


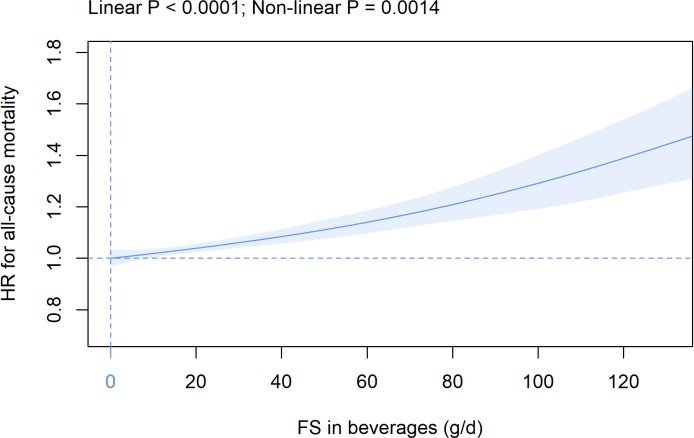

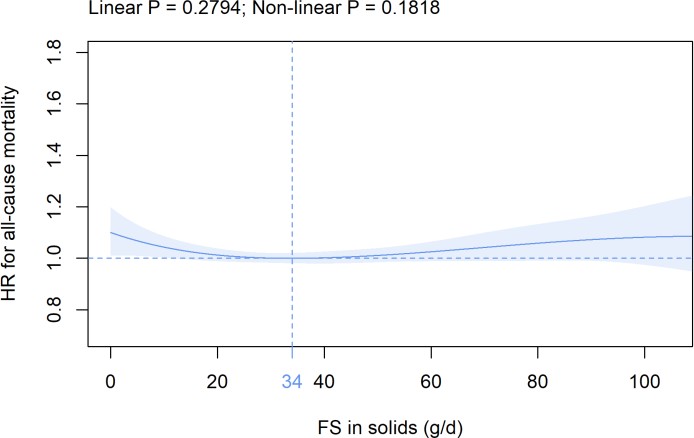


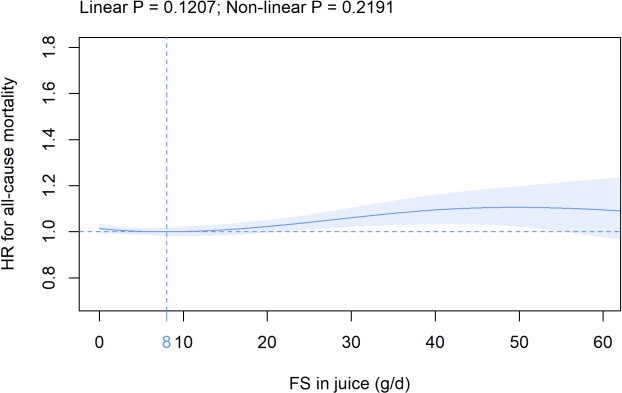
(e) (f)


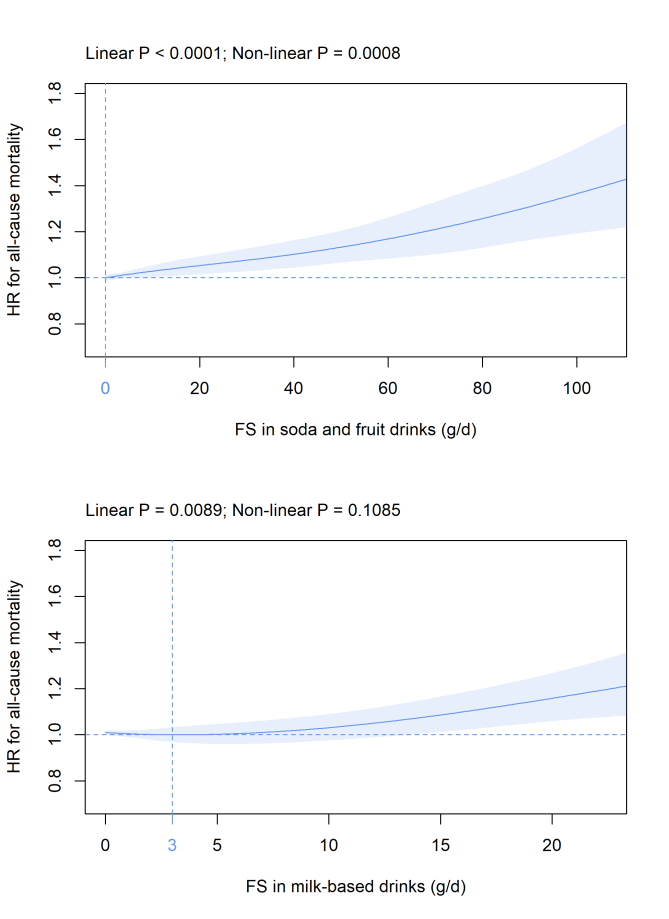

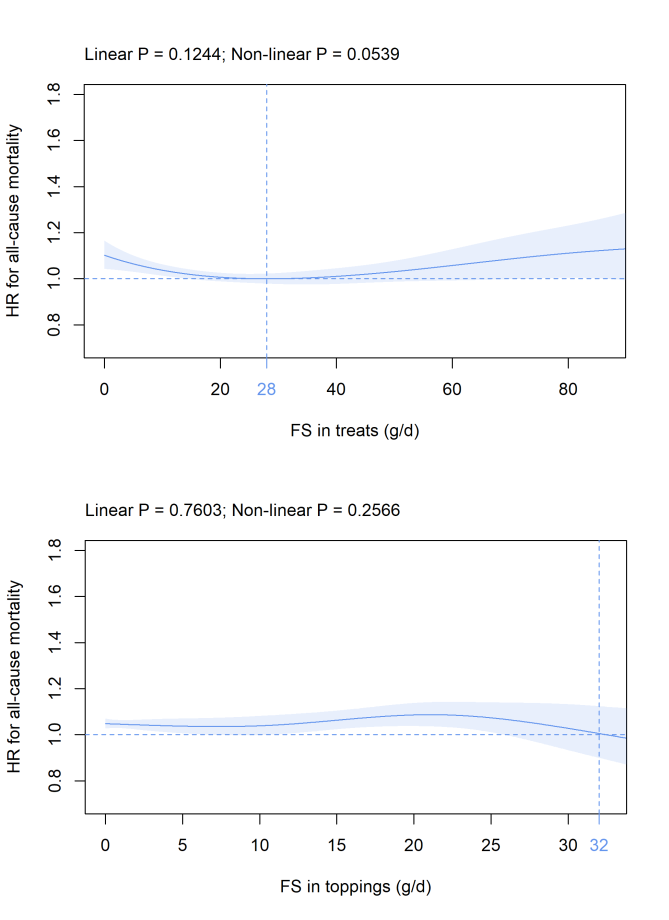


(g) (h)


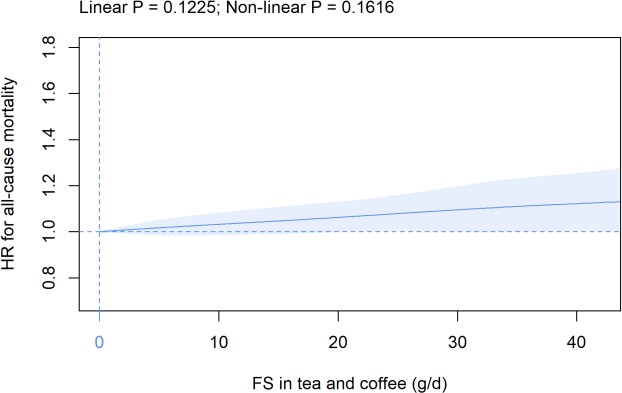


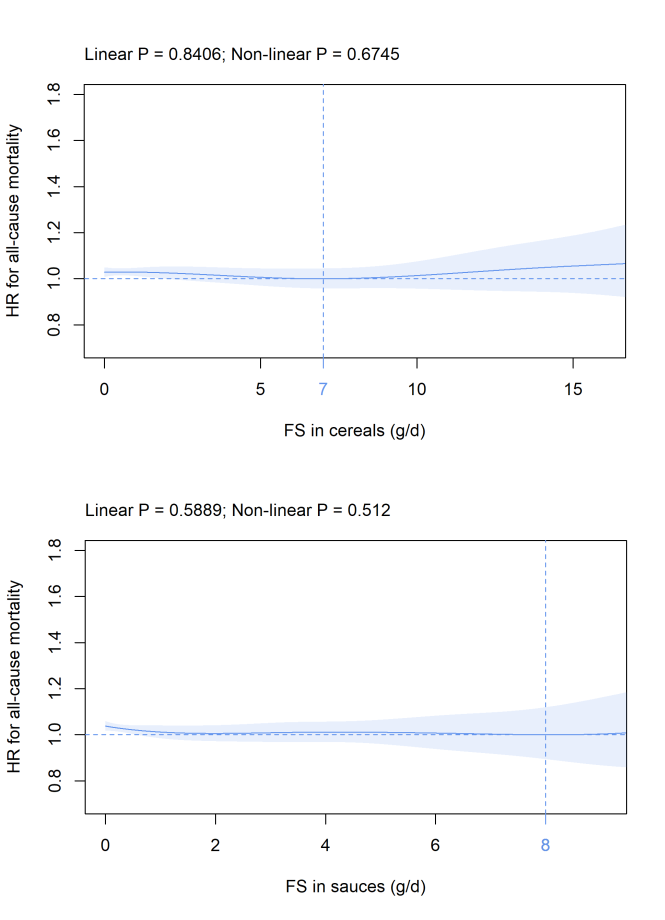
(i) (j)

(k) (l)

(a) (b)


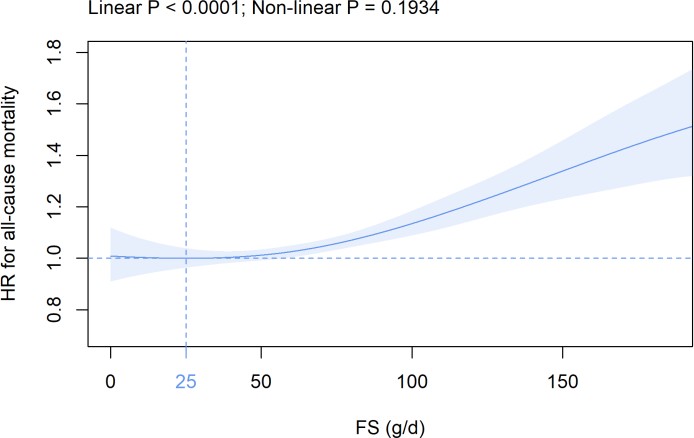

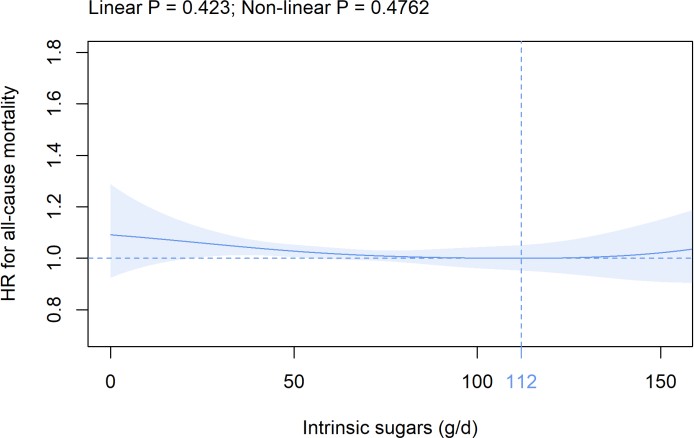


(c) (d)


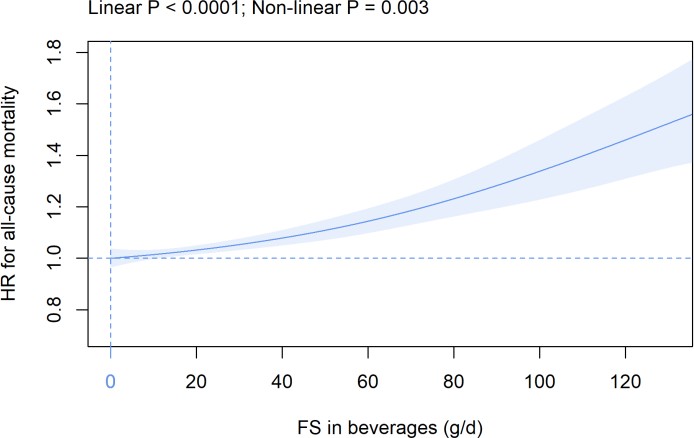

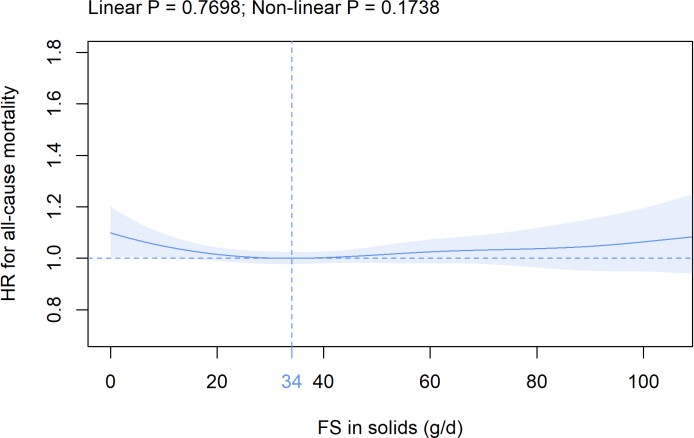


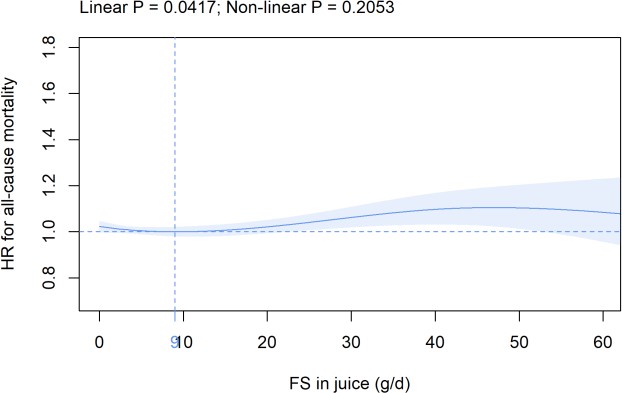
(e) (f)


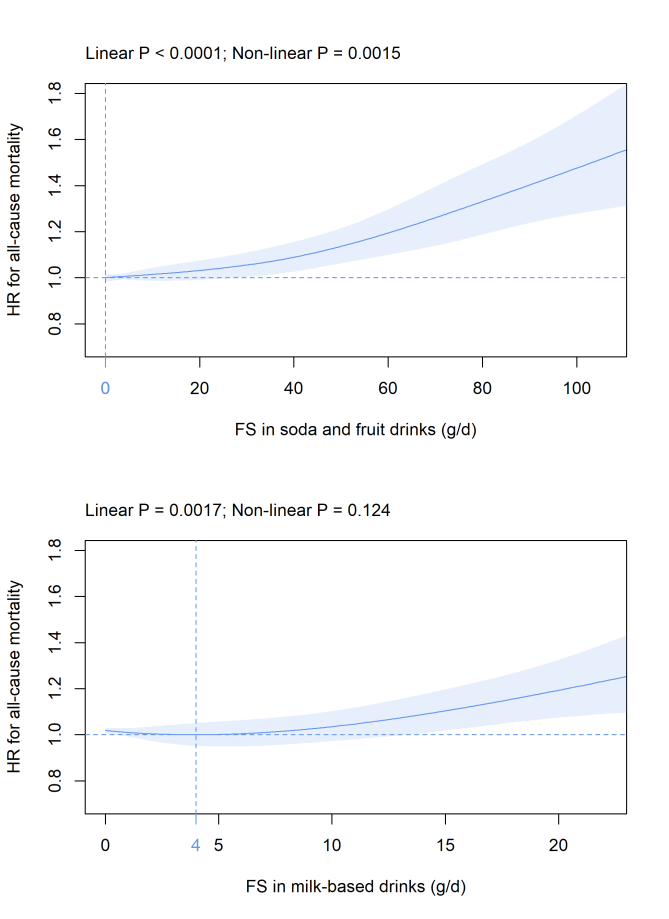

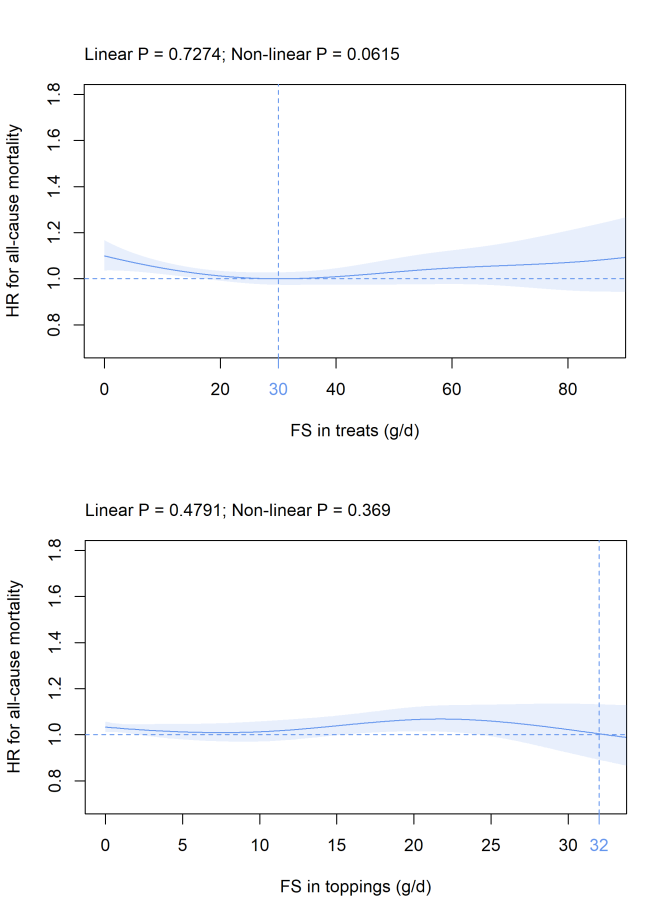


(g) (h)


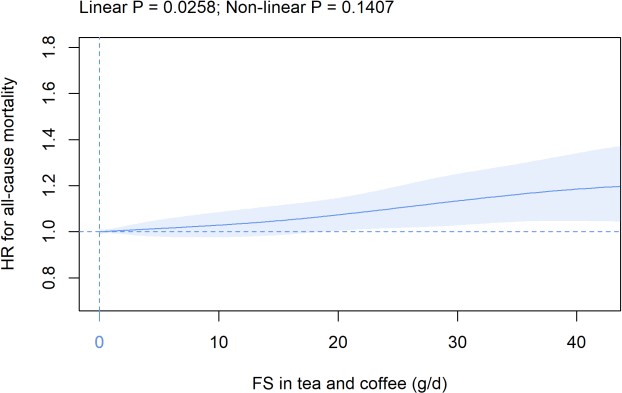


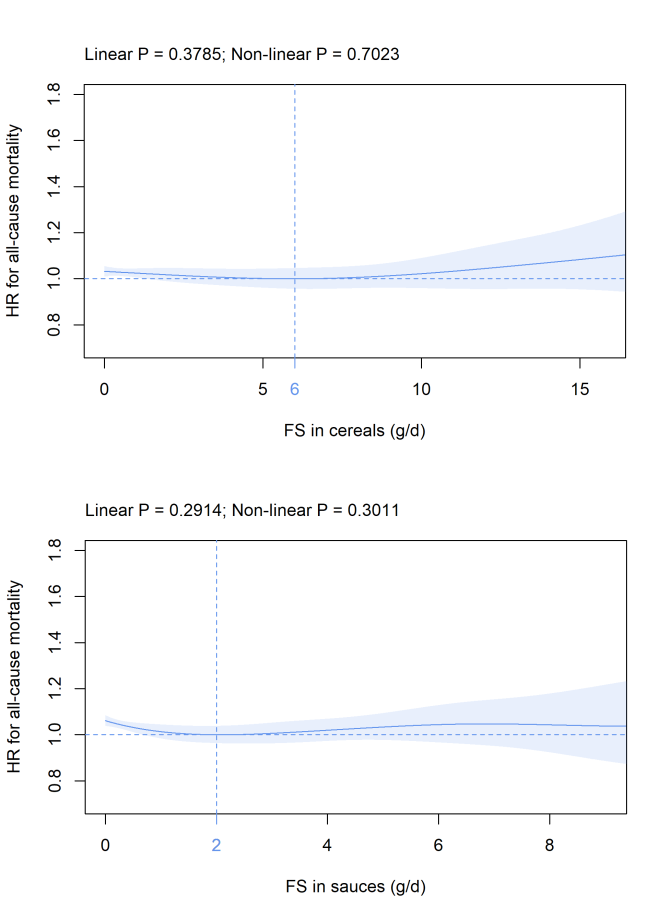
(i) (j)

(k) (l)

(a) (b)


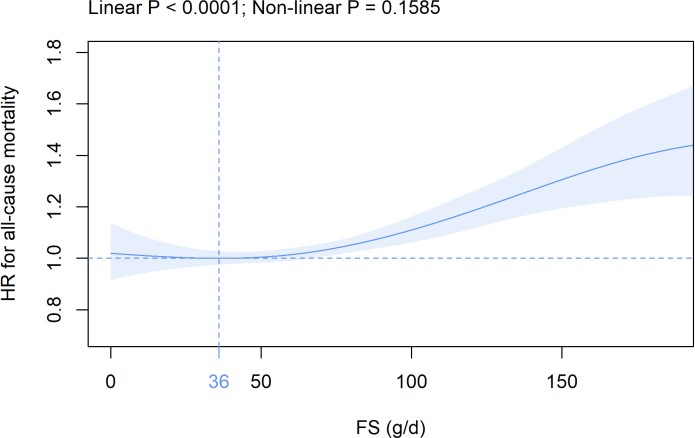

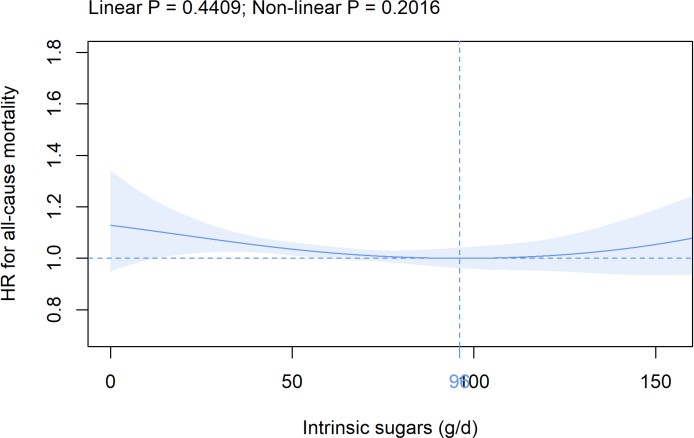


(c) (d)


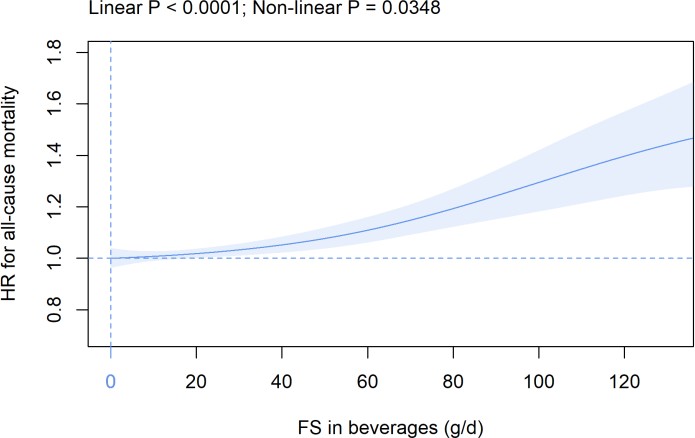

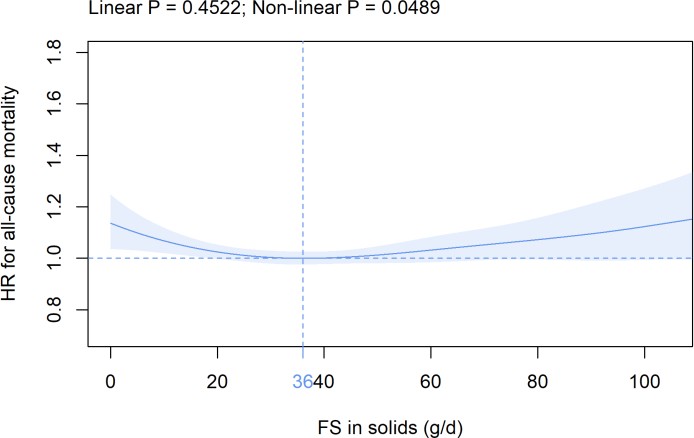


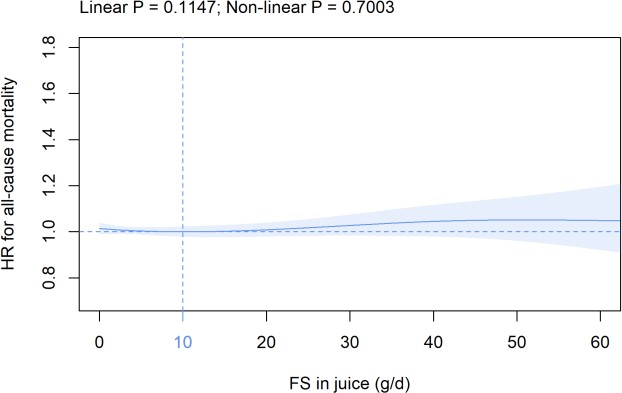
(e) (f)


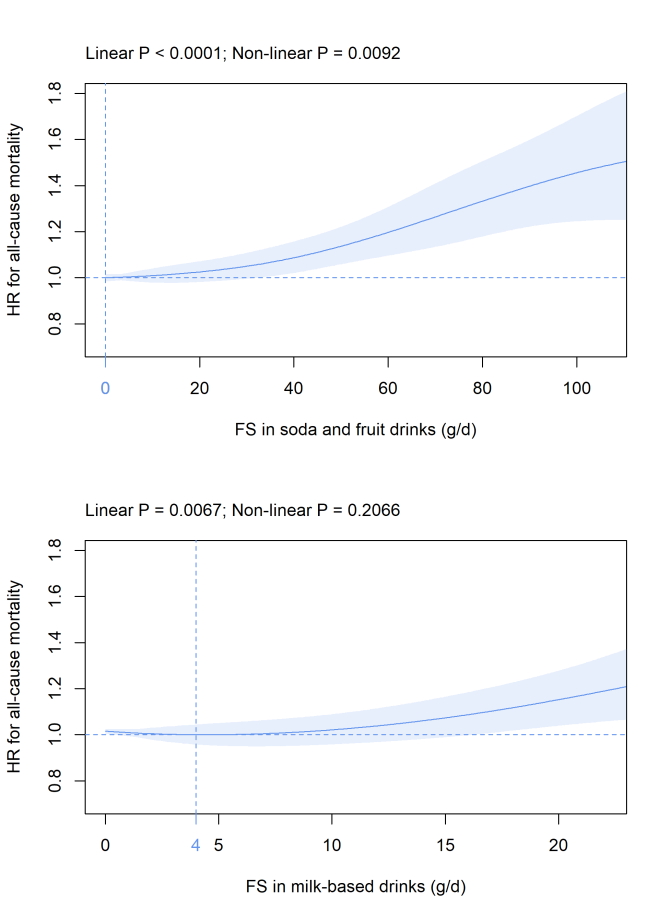

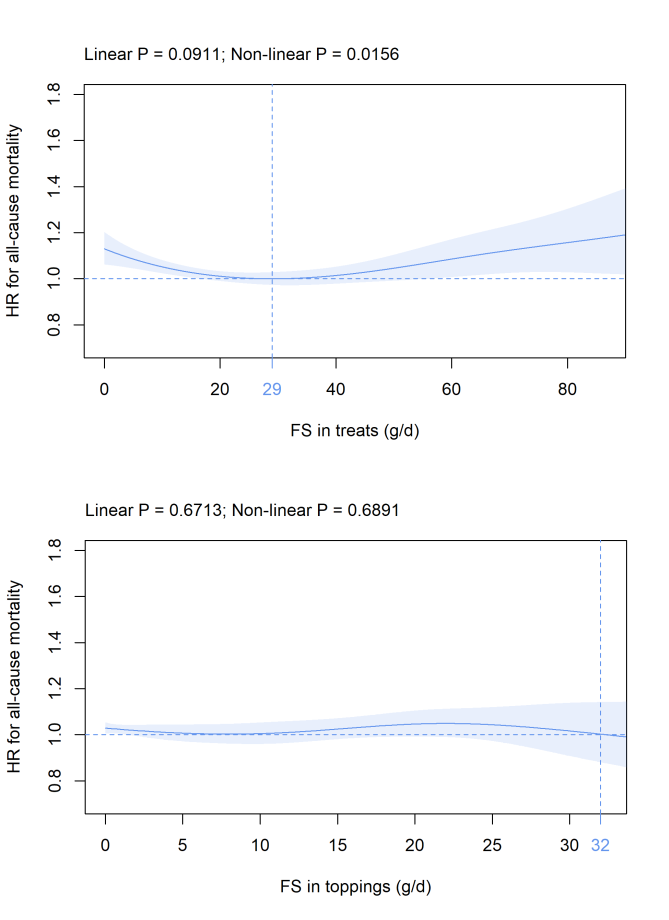


(g) (h)


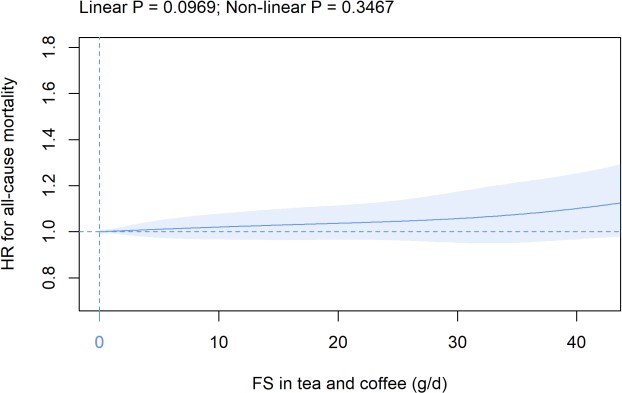


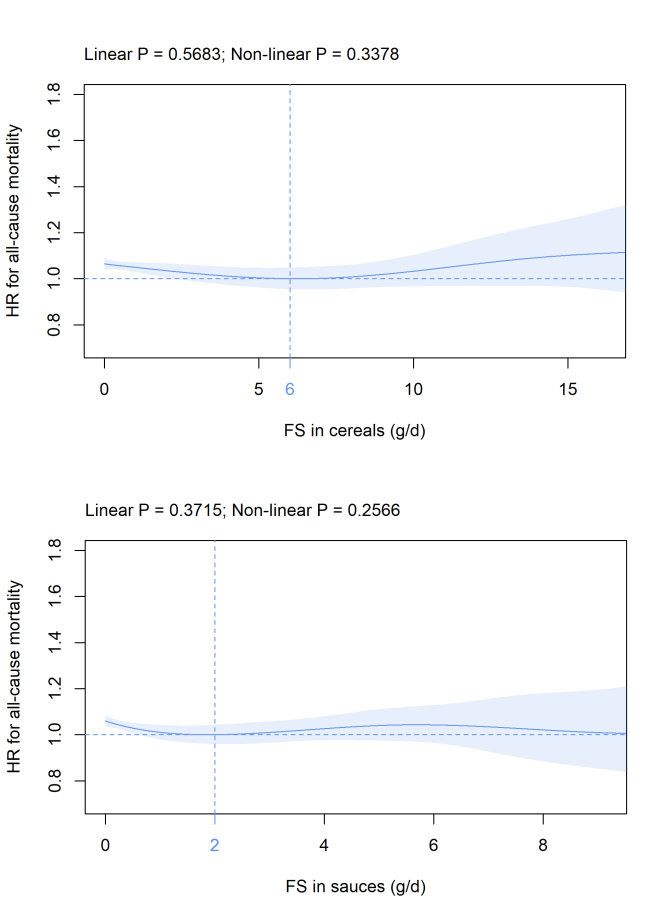
(i) (j)

(k) (l)

(a) (b)


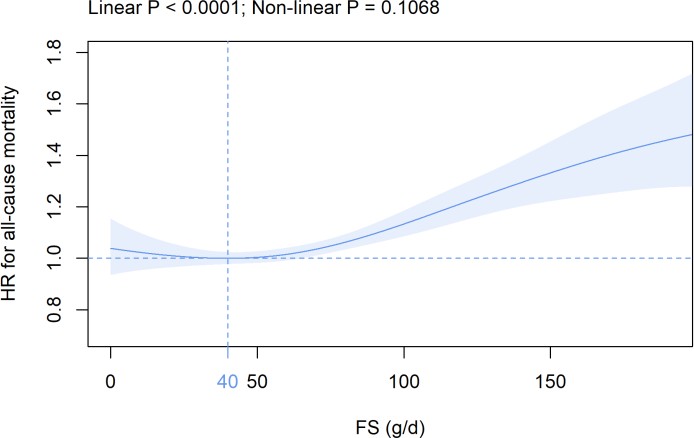

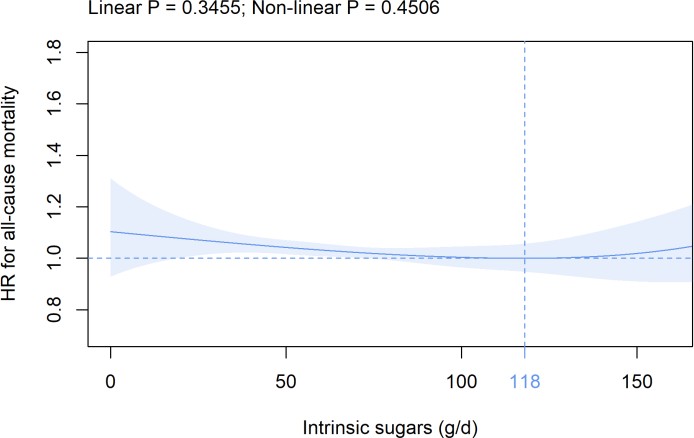


(c) (d)


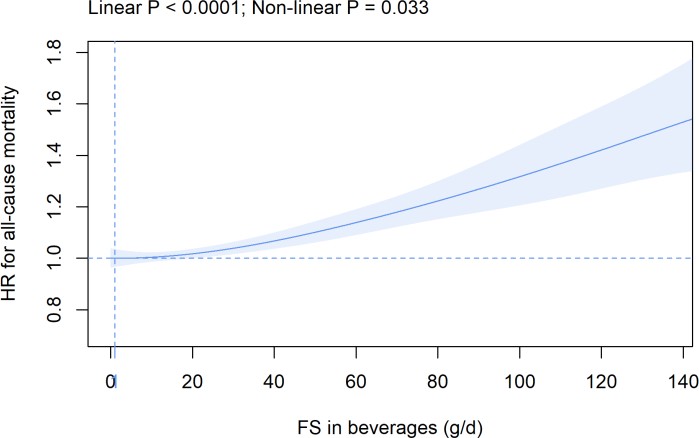

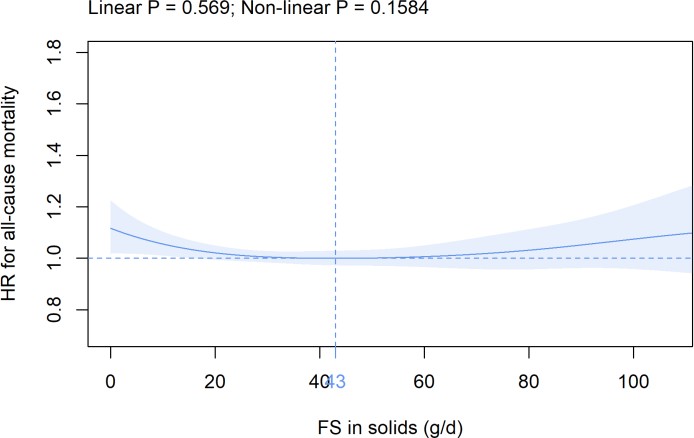


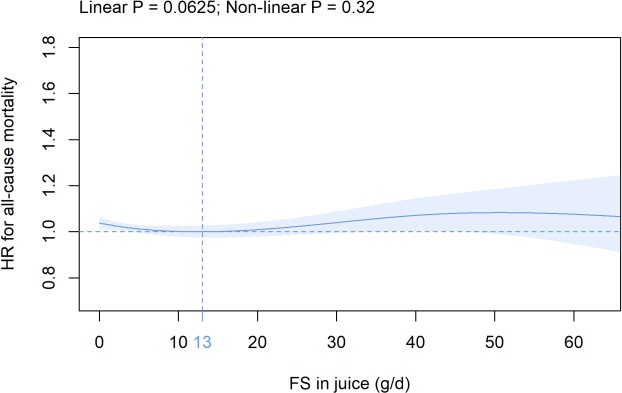
(e) (f)


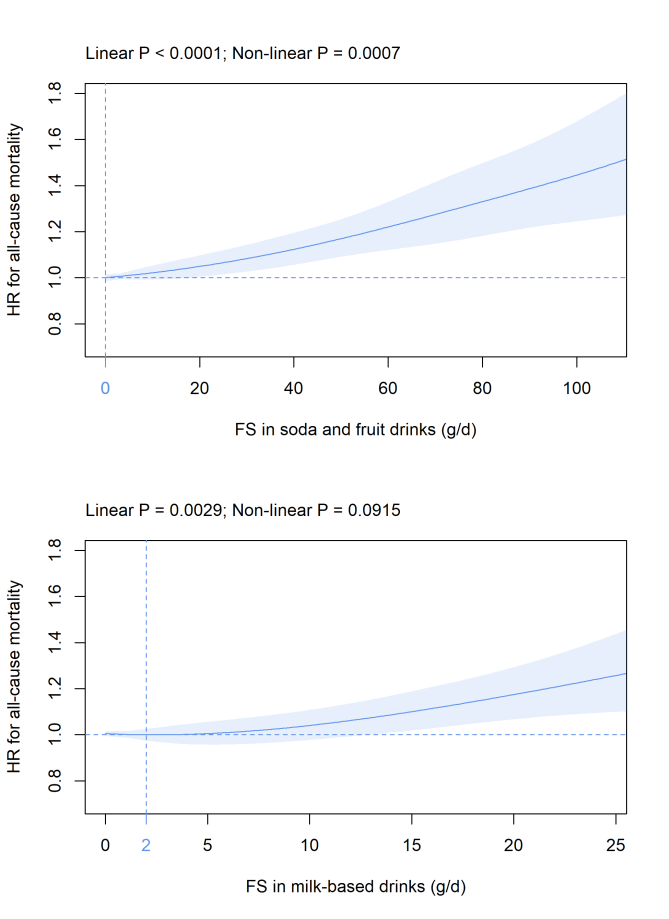

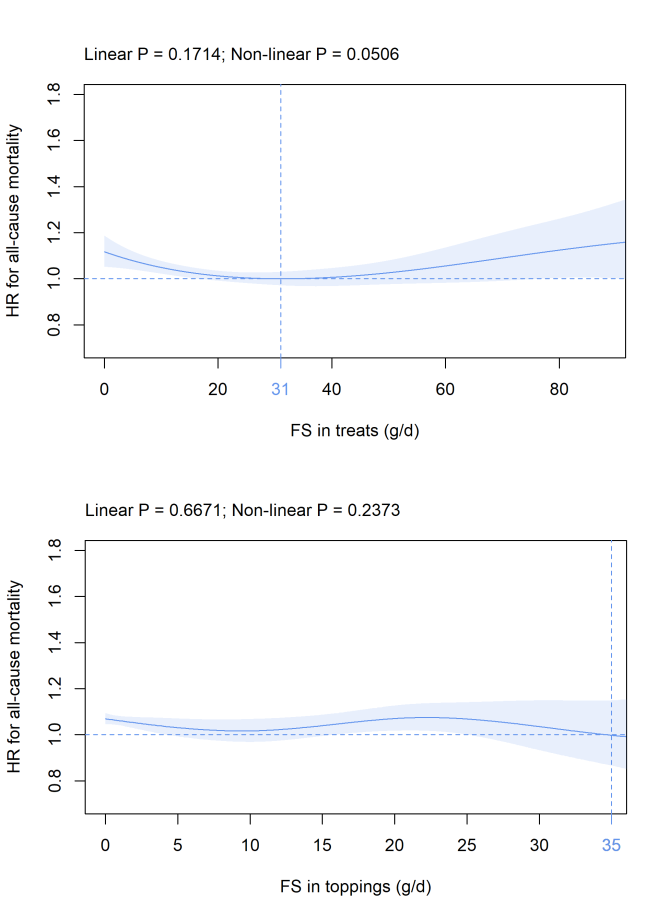


(g) (h)


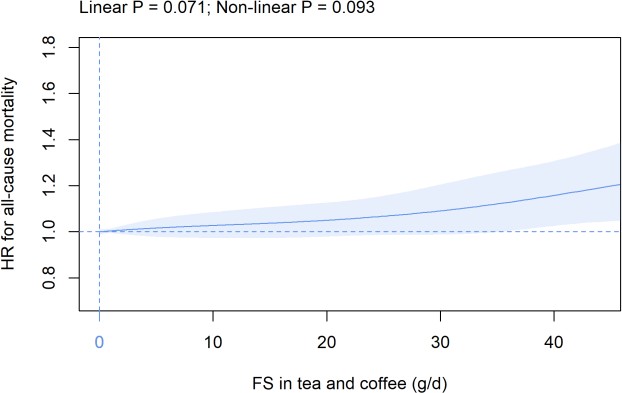


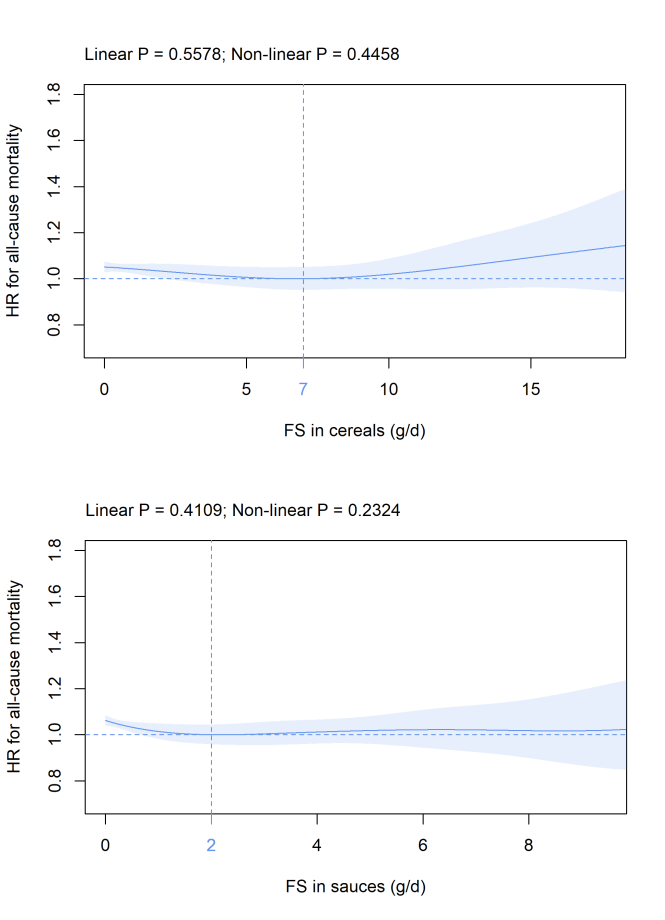
(i) (j)

(k) (l)

(a) (b)


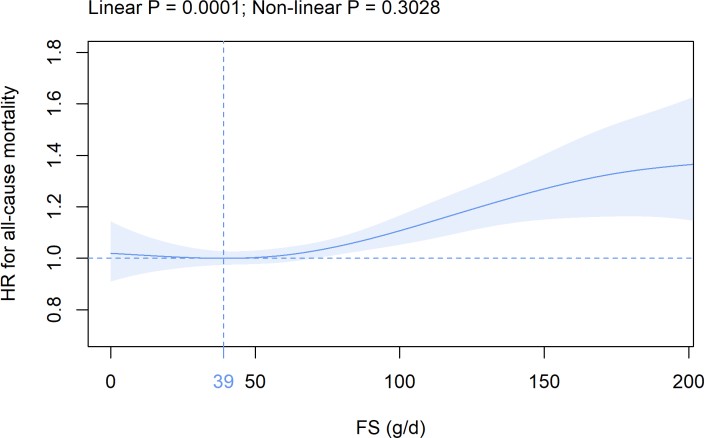

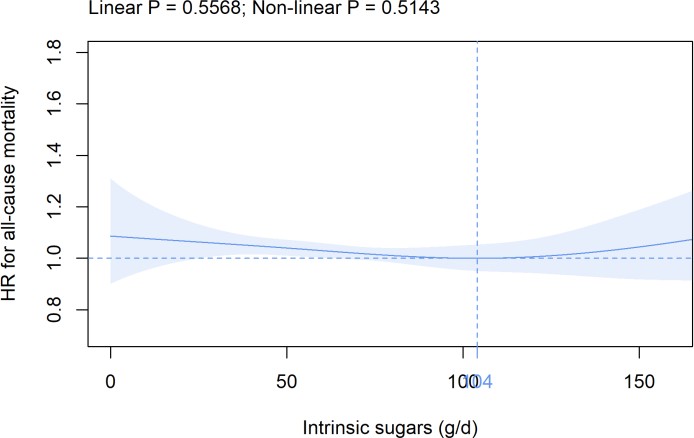


(c) (d)


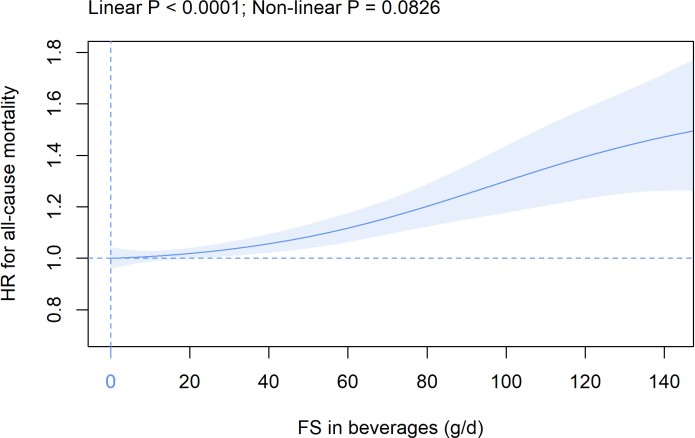

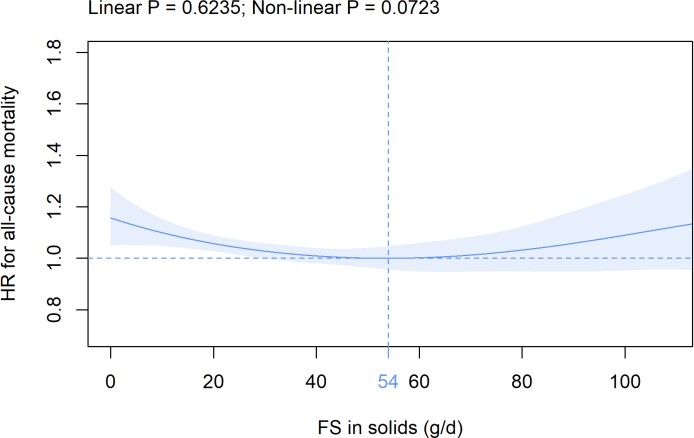


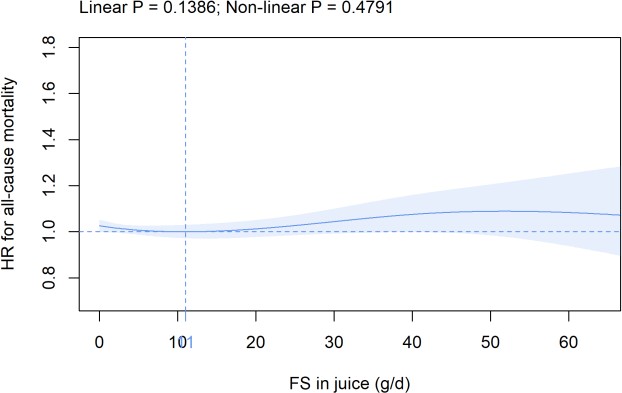
(e) (f)


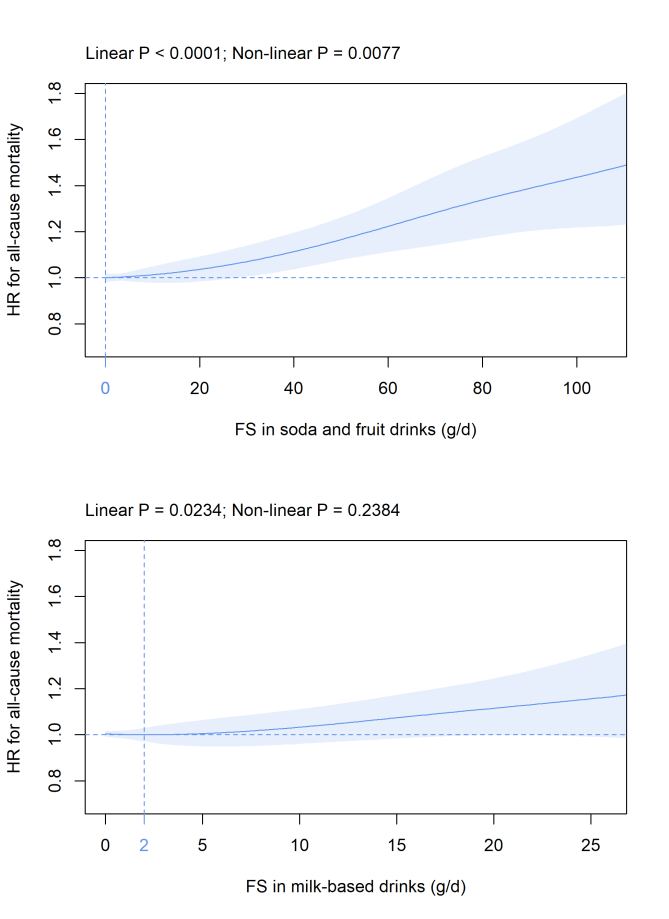

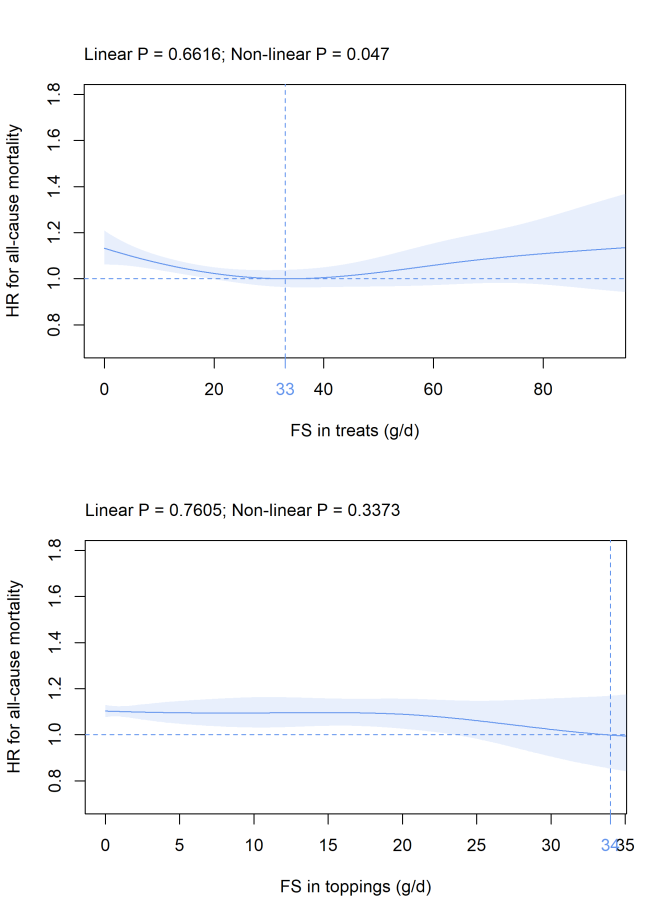


(g) (h)


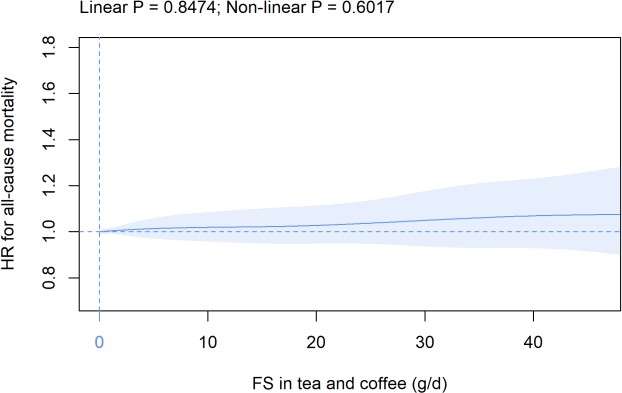


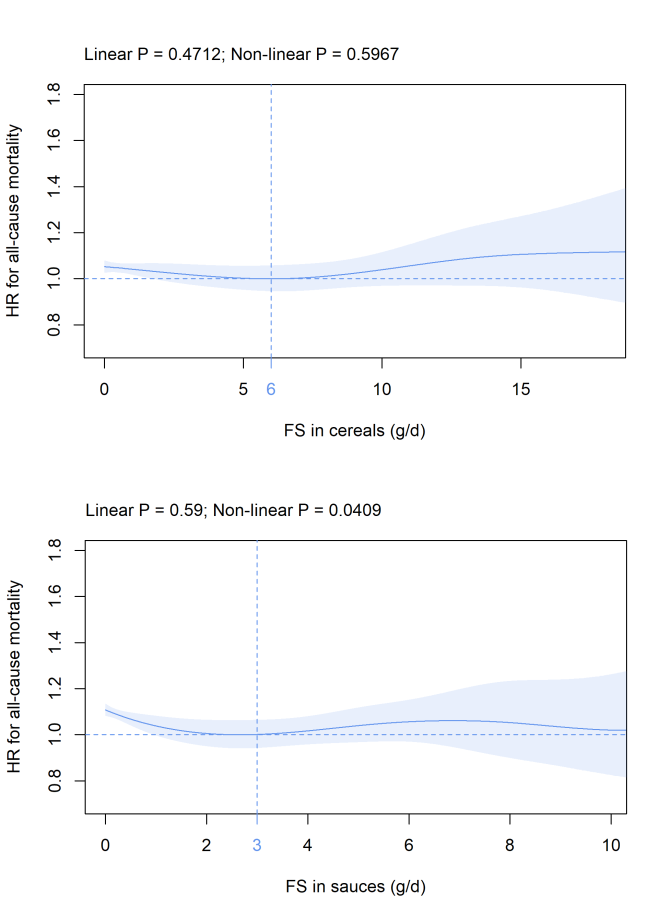
(i) (j)

(k) (l)

(a) (b)


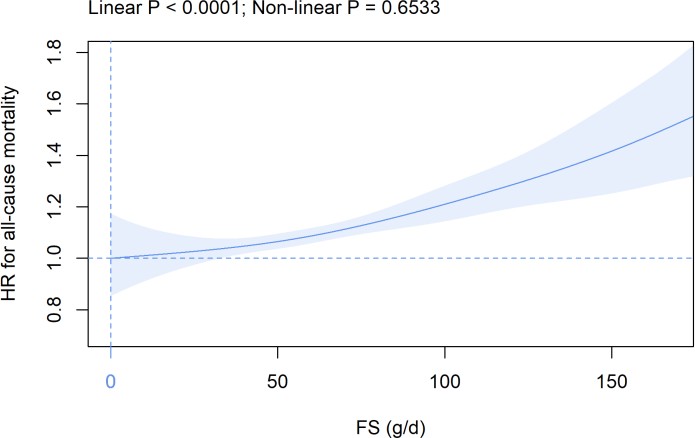

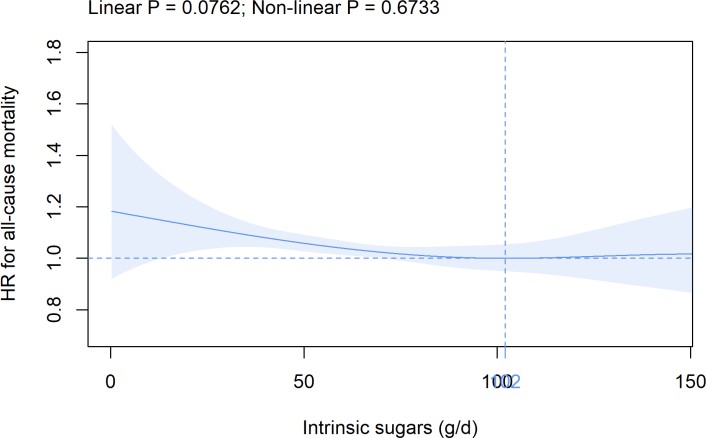


(c) (d)


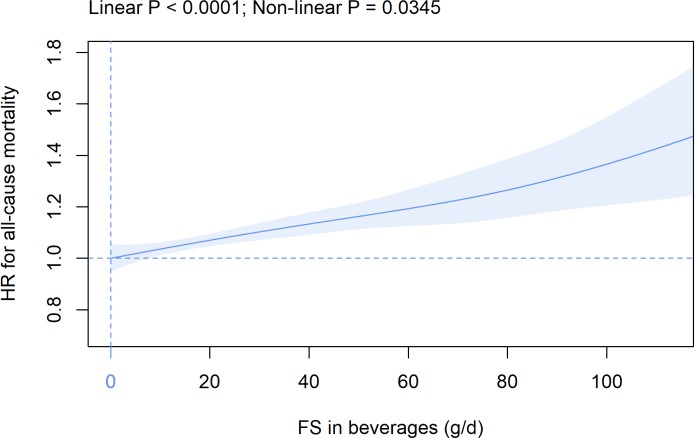

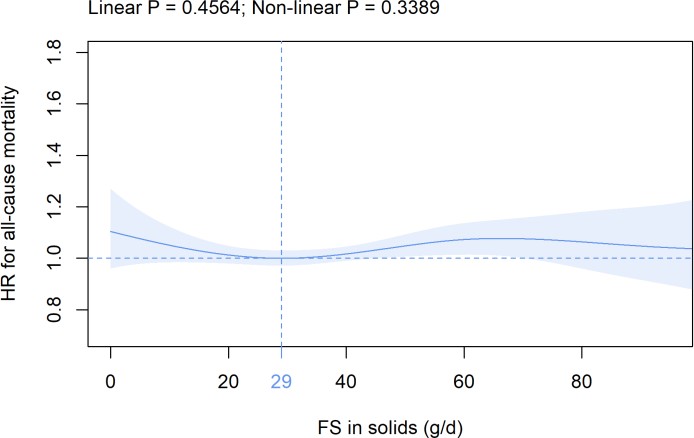


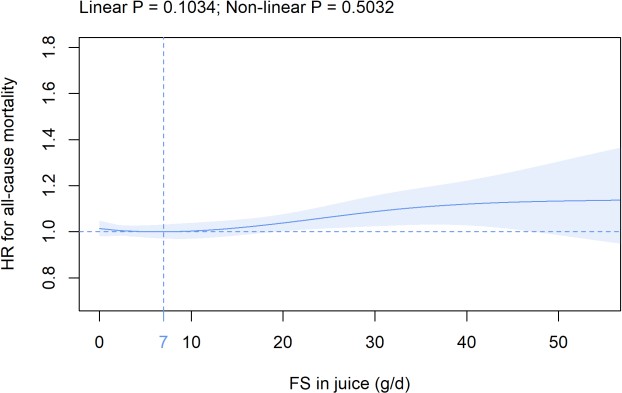
(e) (f)


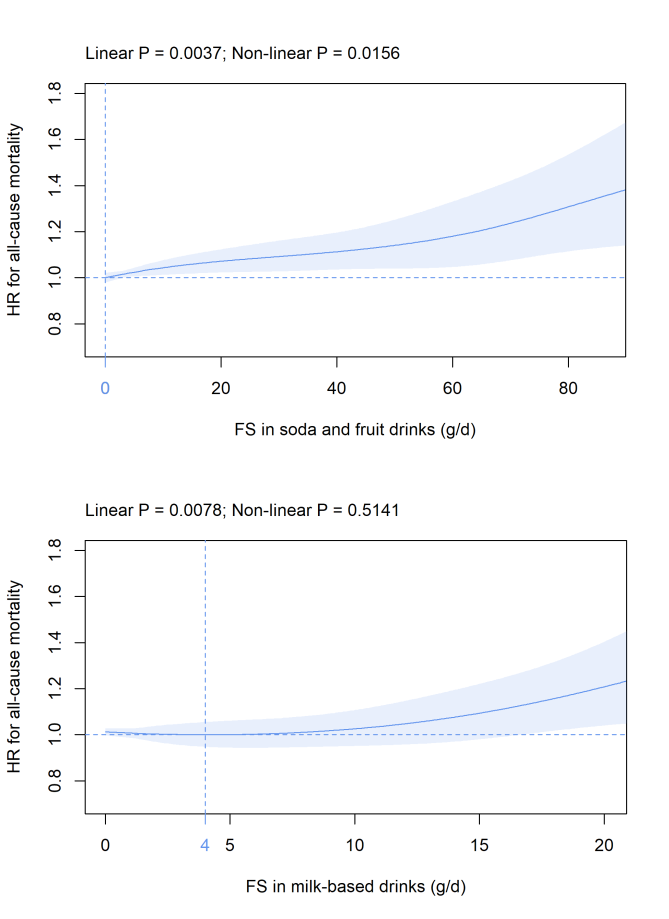

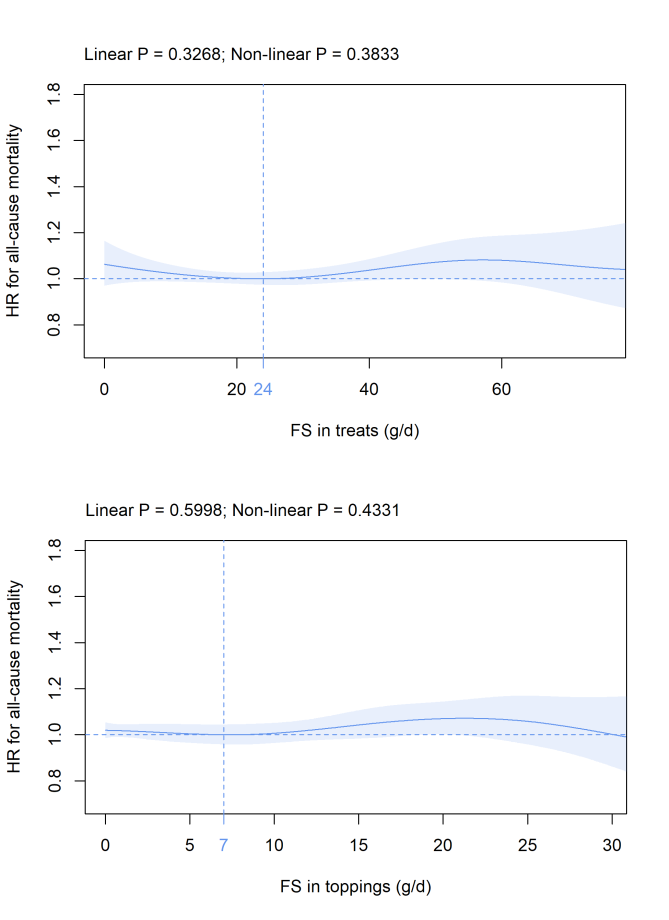


(g) (h)


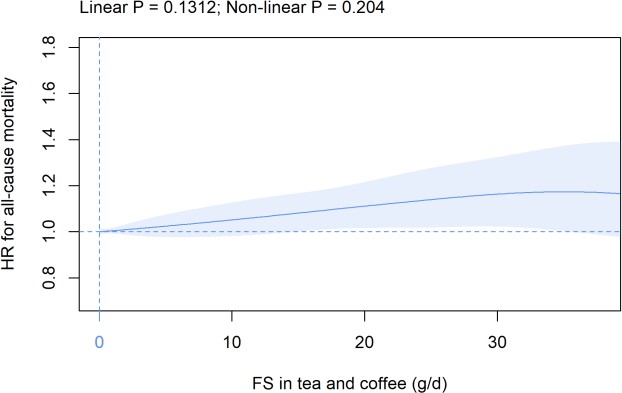


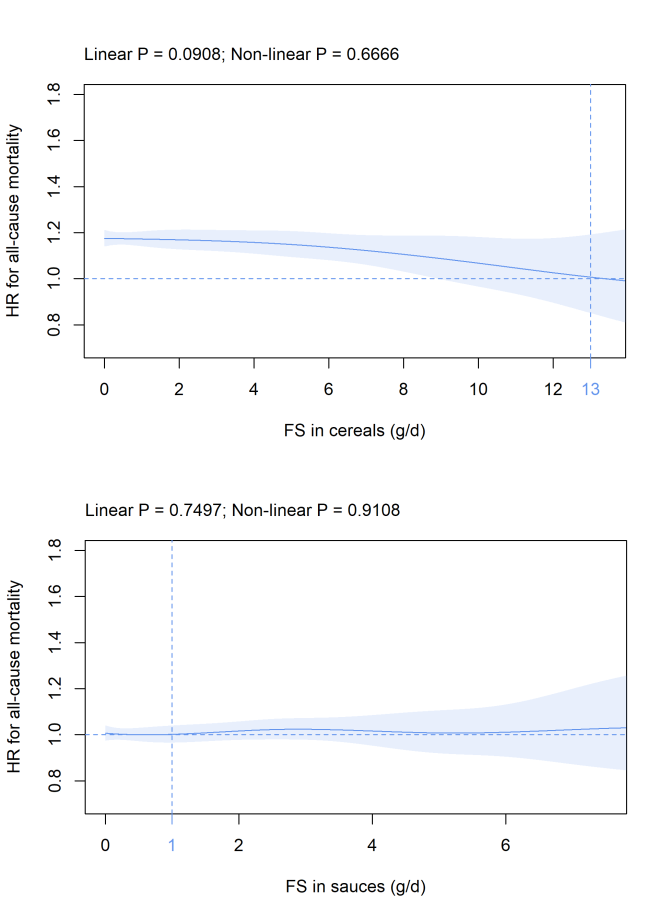
(i) (j)

(k) (l)

(a) (b)


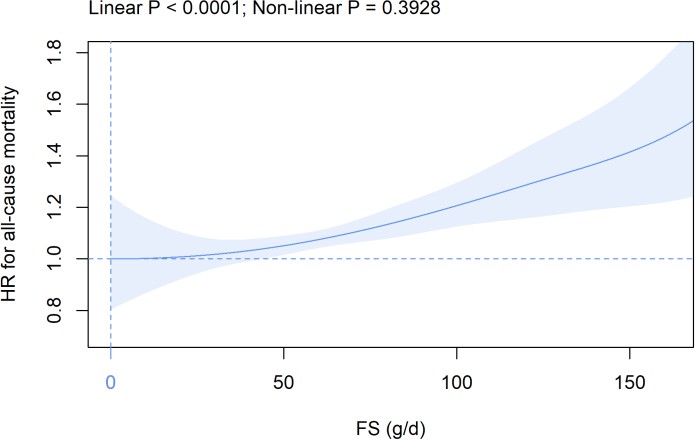

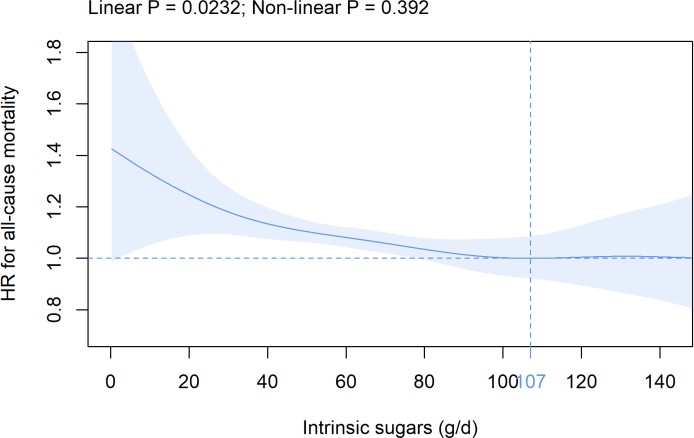


(c) (d)


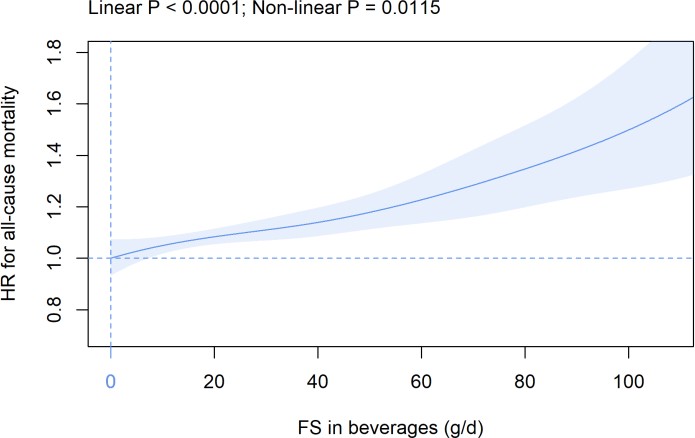

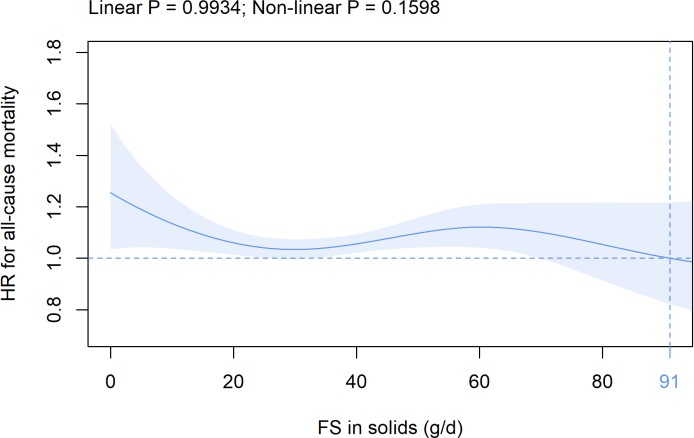


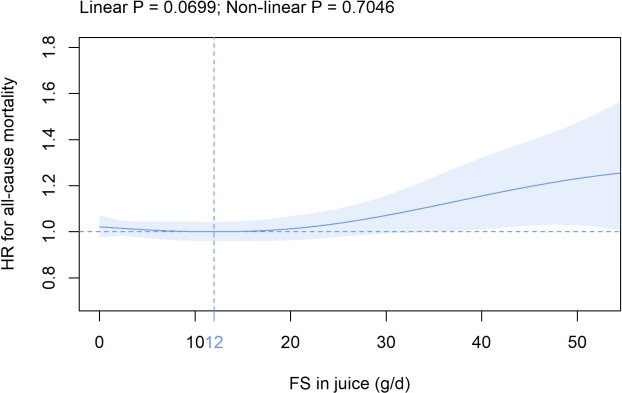
(e) (f)


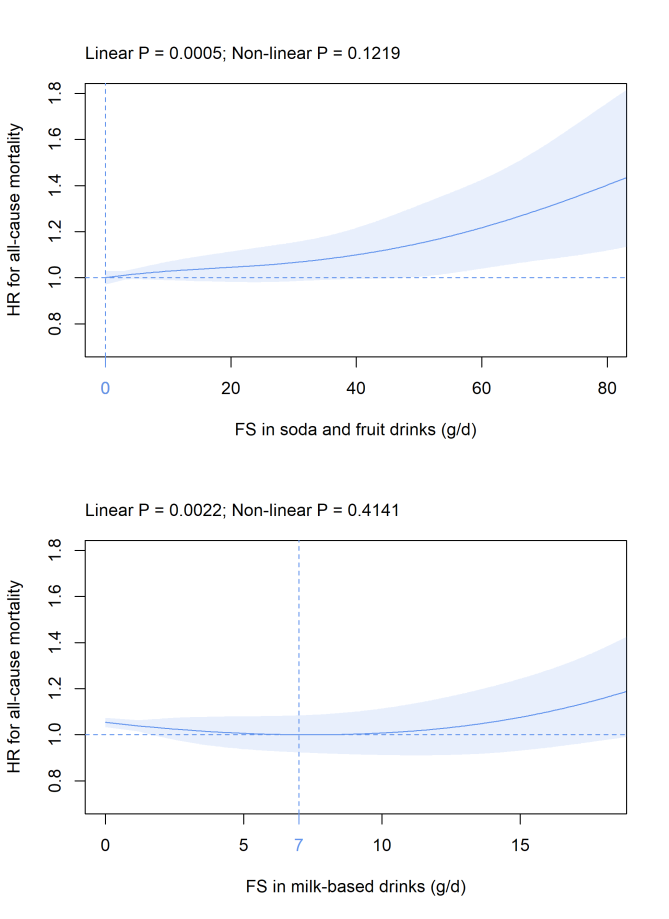

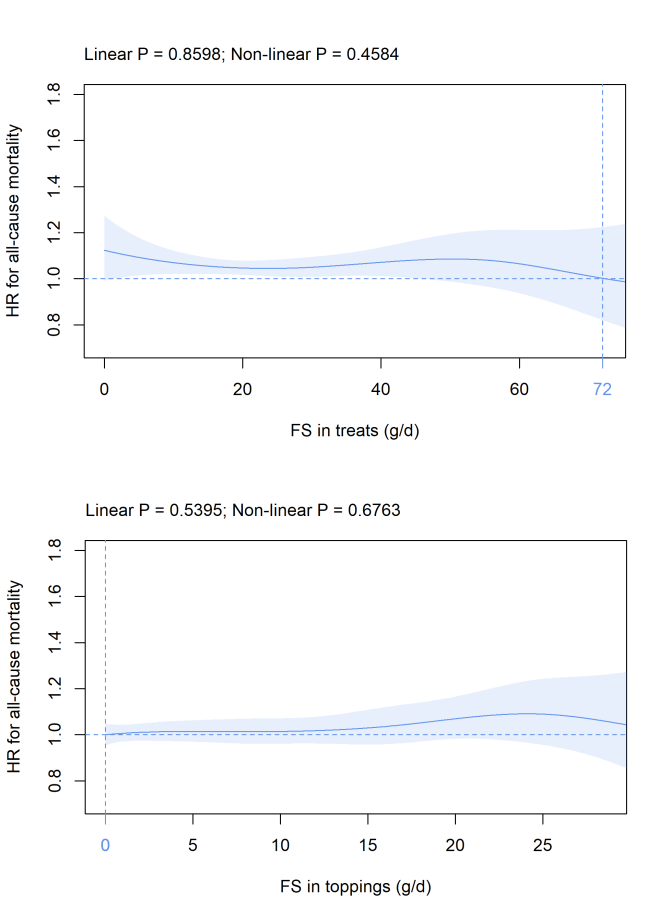


(g) (h)


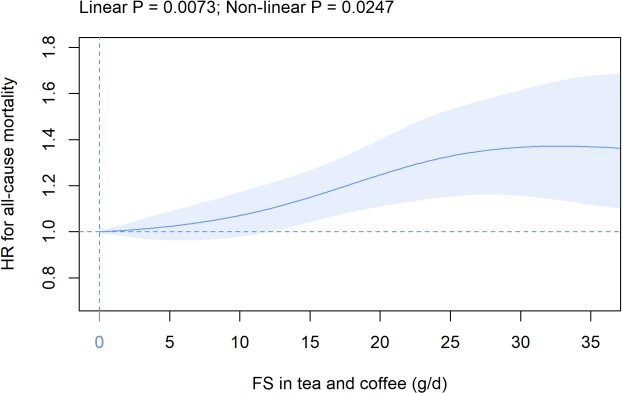


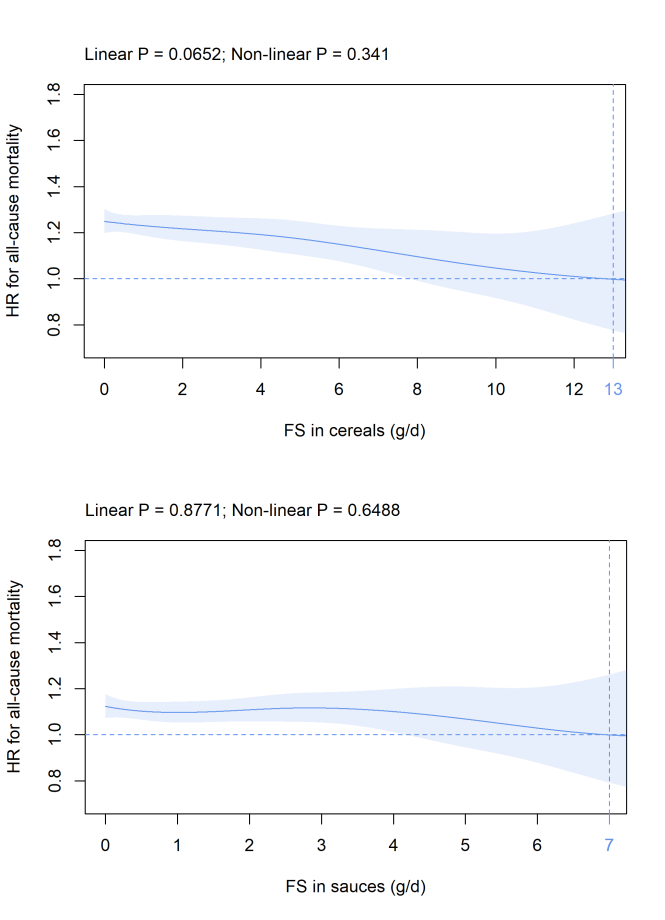
(i) (j)

(k) (l)

(a) (b)


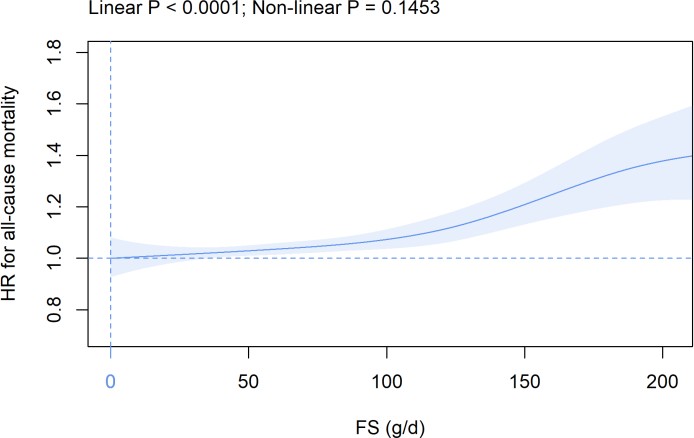

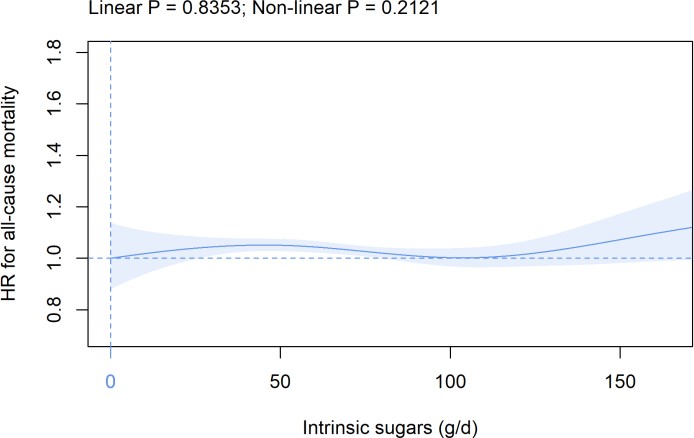


(c) (d)


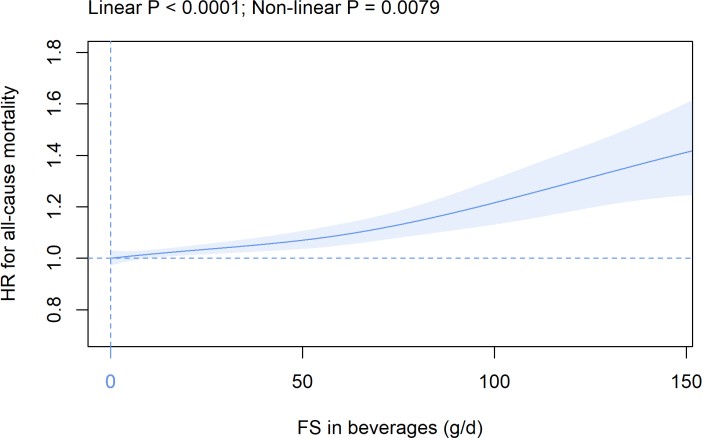

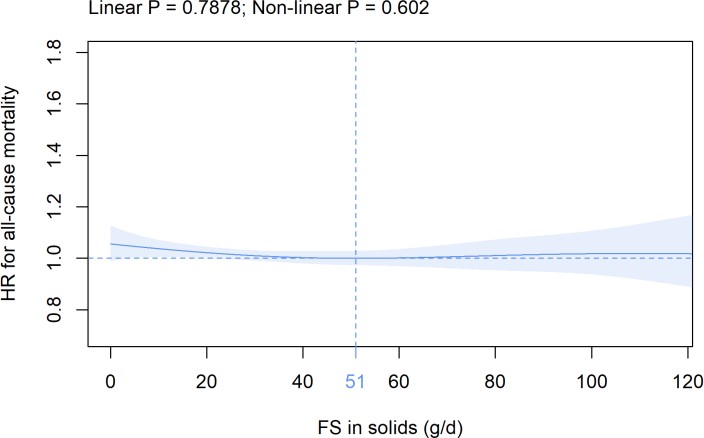


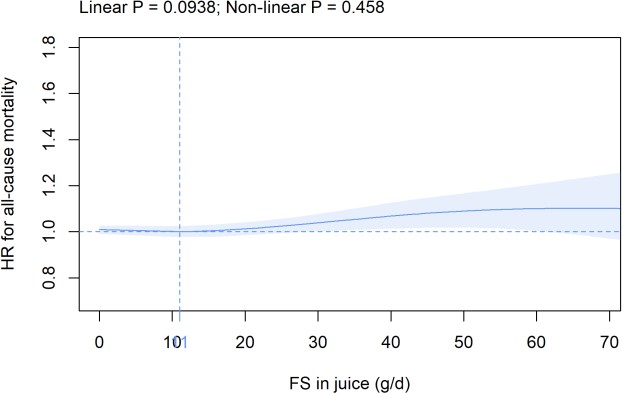
(e) (f)


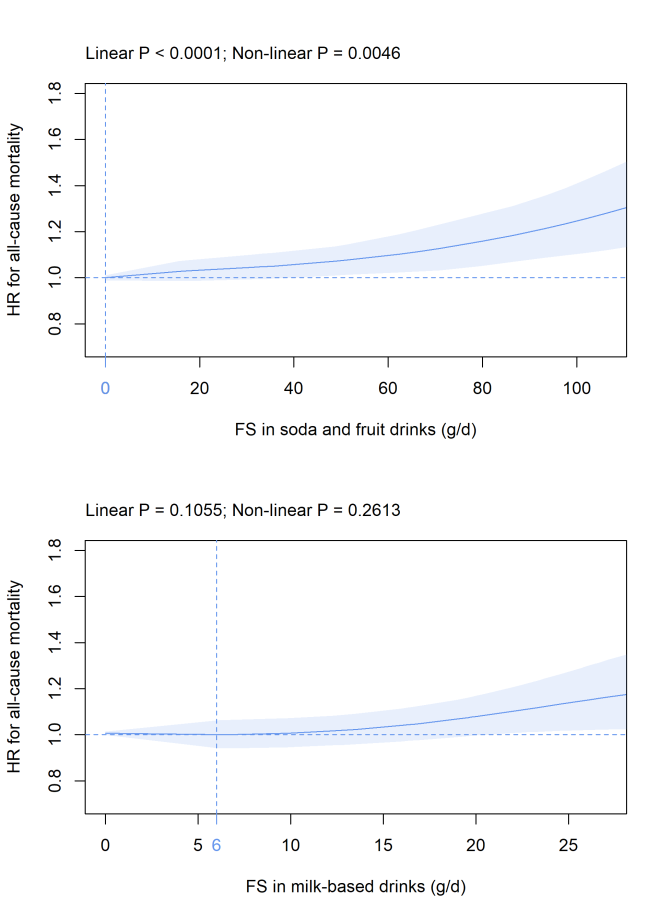

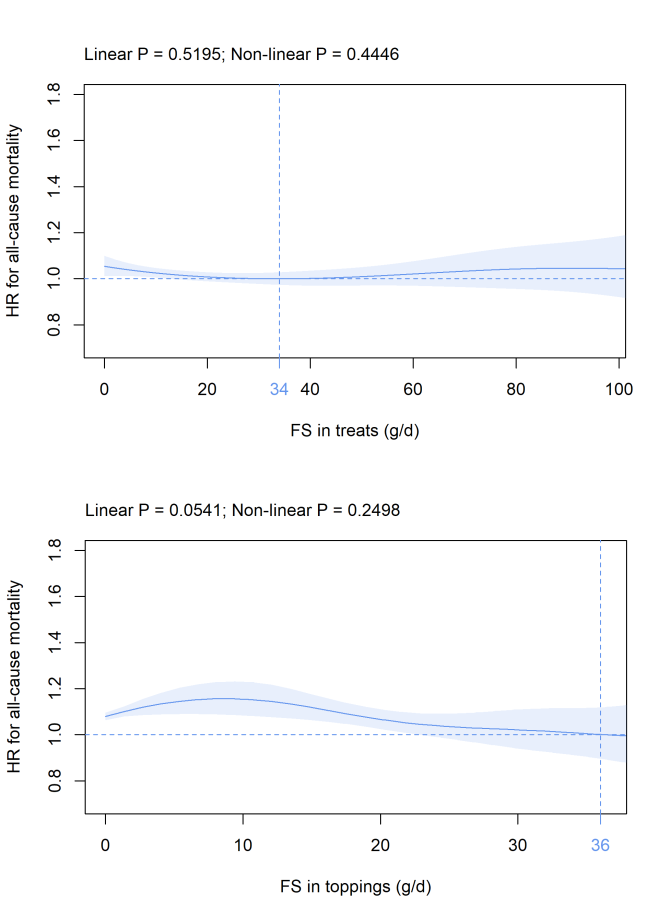


(g) (h)


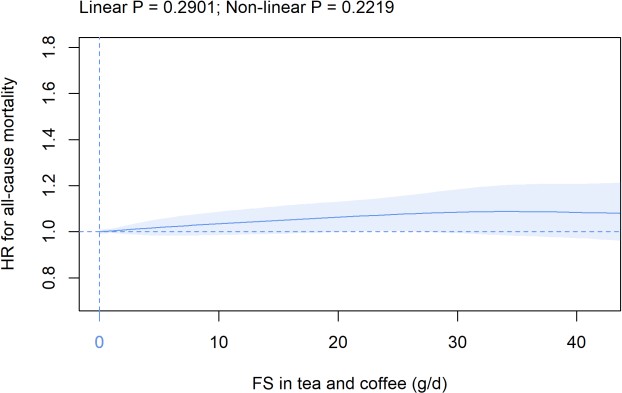


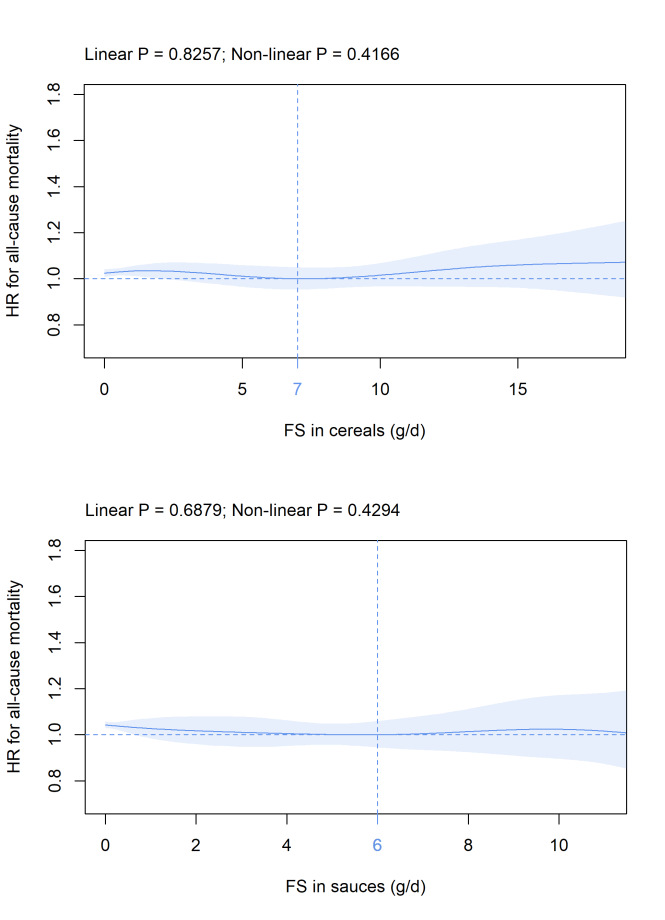
(i) (j)

(k) (l)

(a) (b)


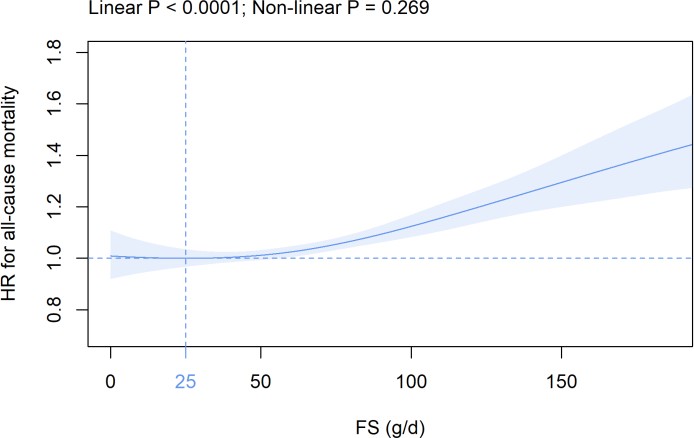

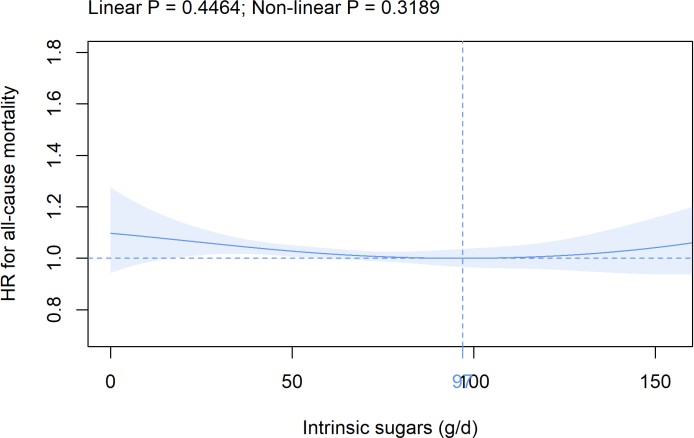


(c) (d)


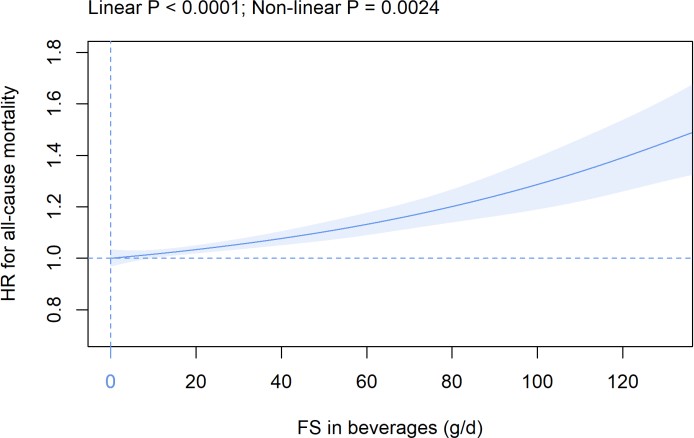

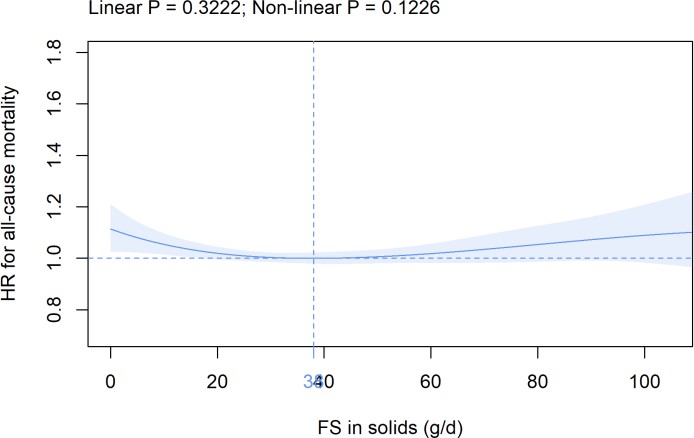


(e) (f)


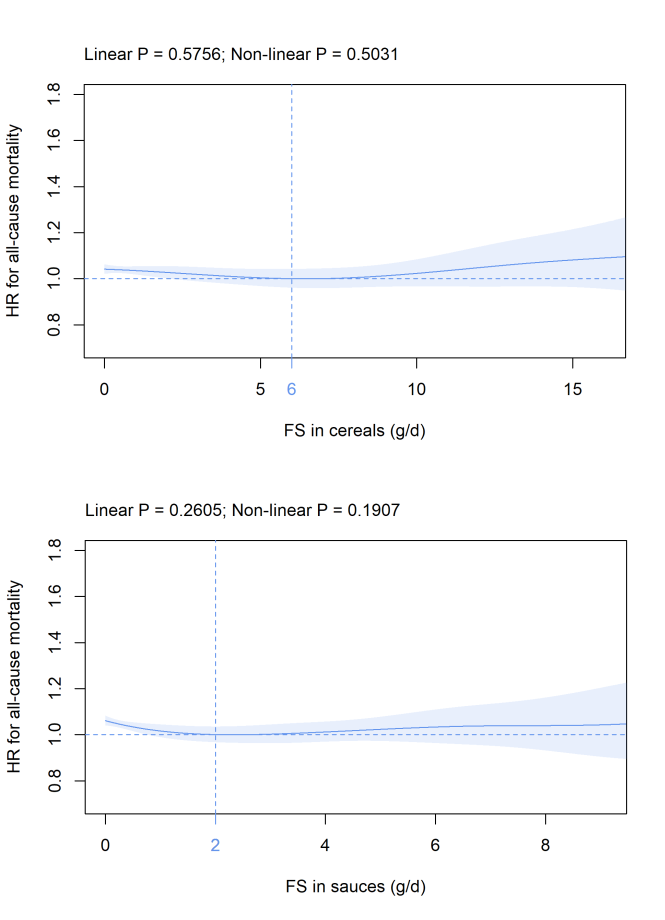

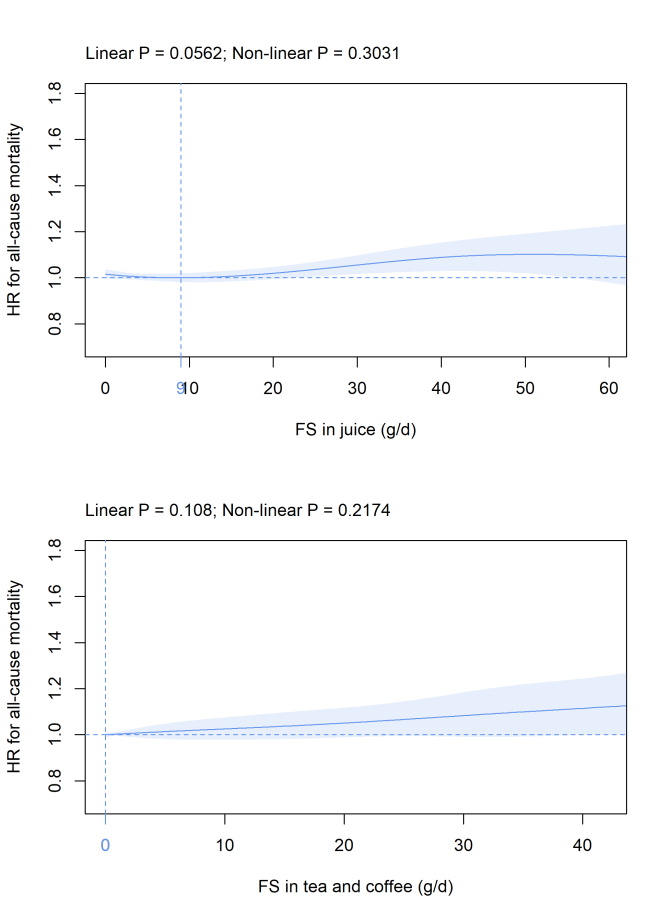

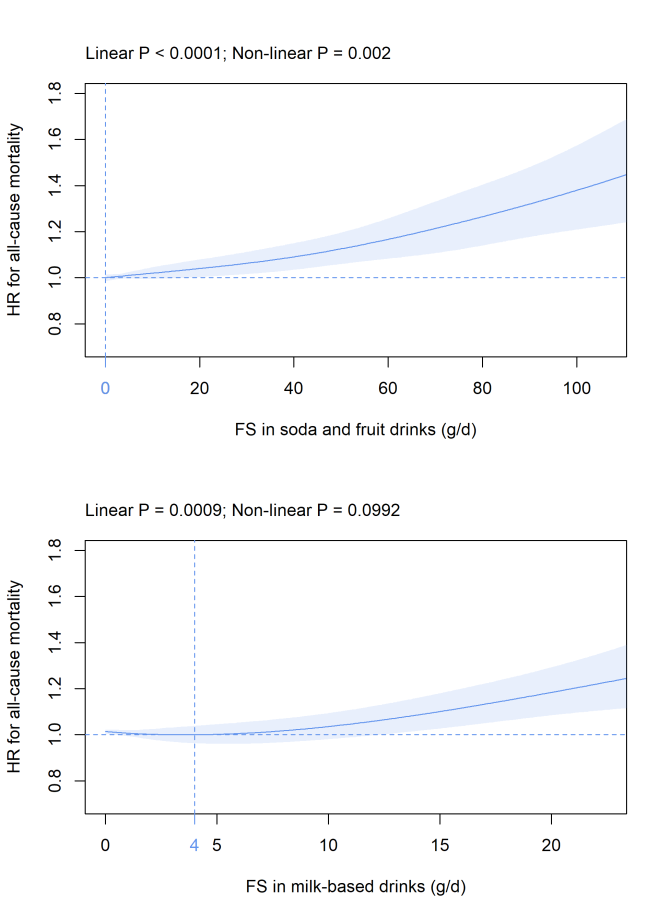

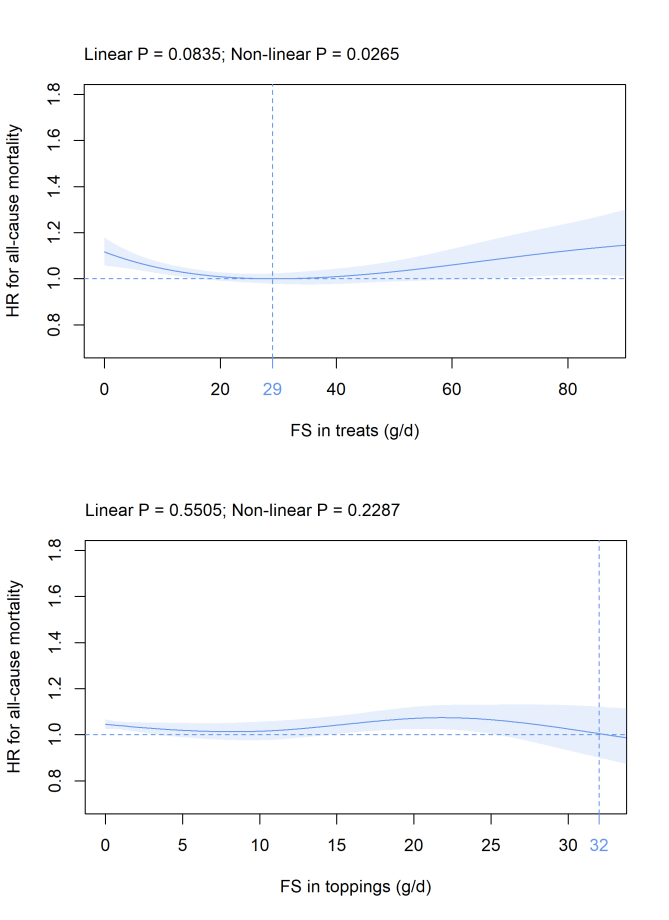


(g) (h)

(i) (j)

(k) (l)

(a) (b)


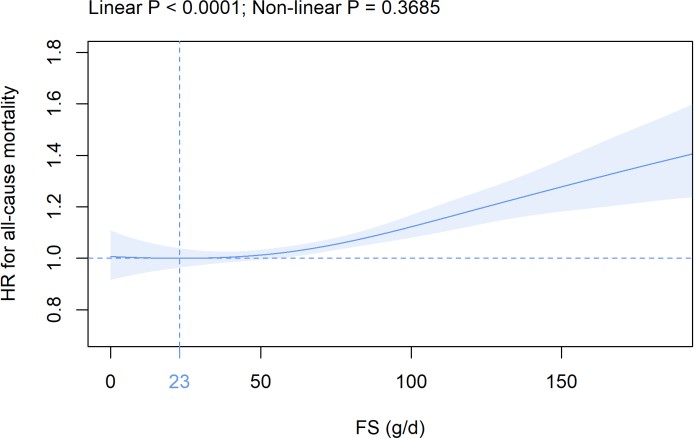

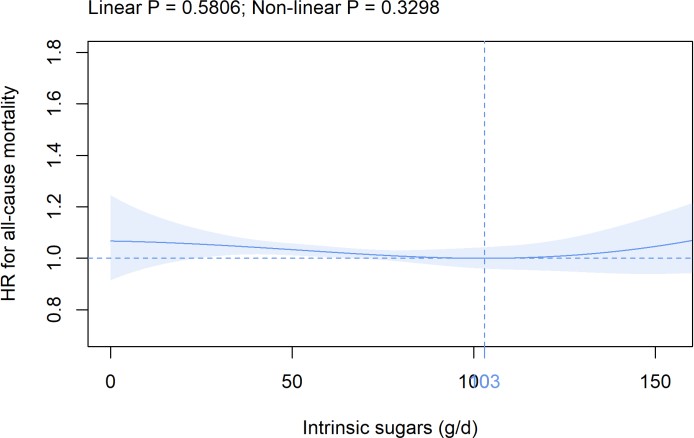


(c) (d)


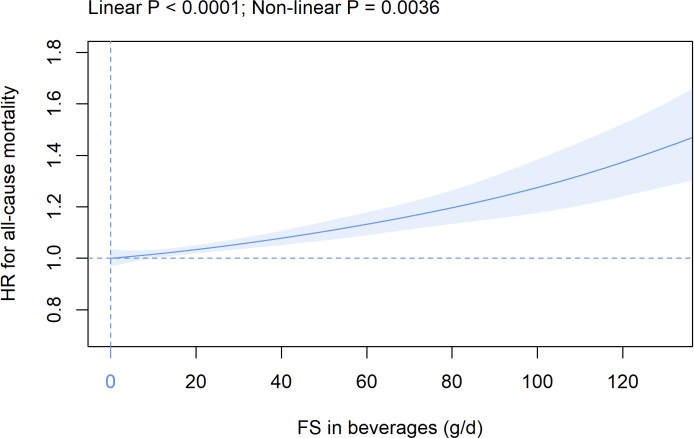

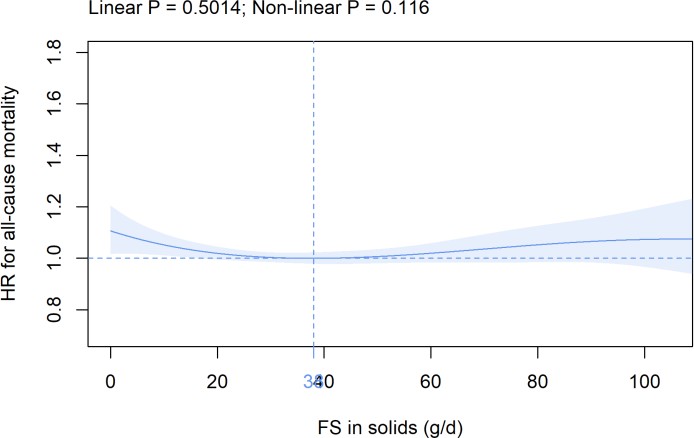


(e) (f)


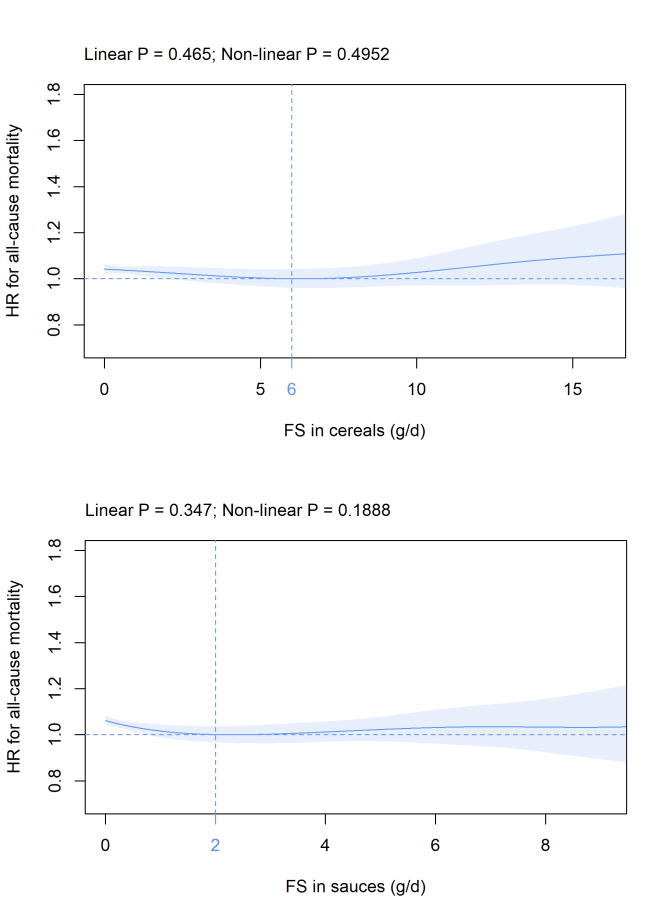

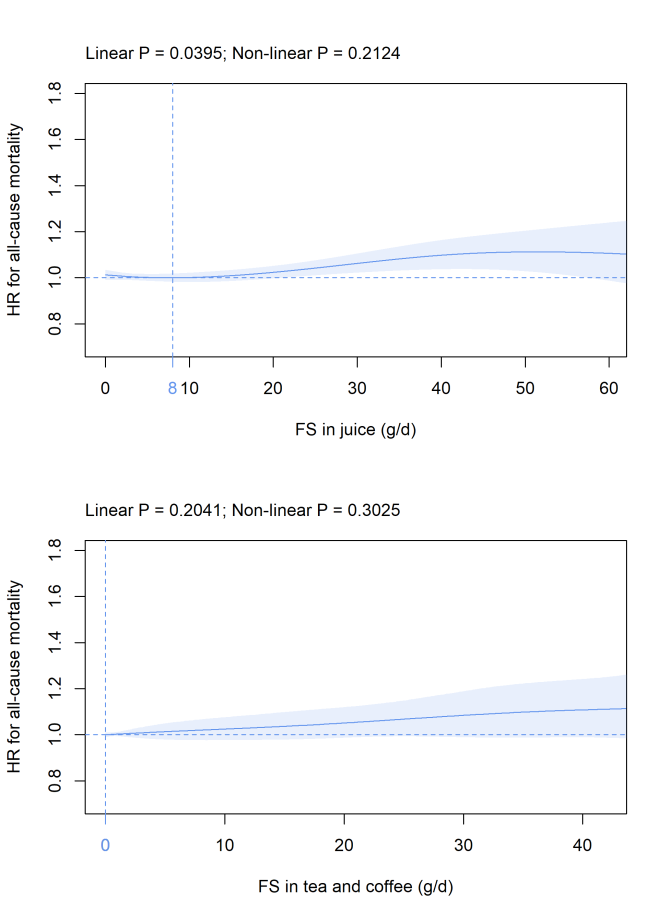

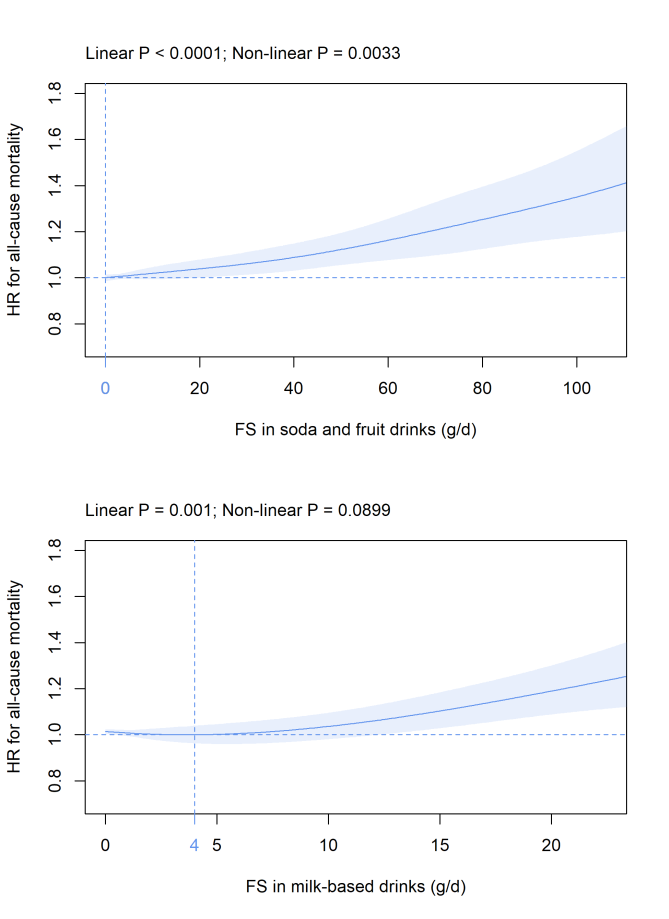

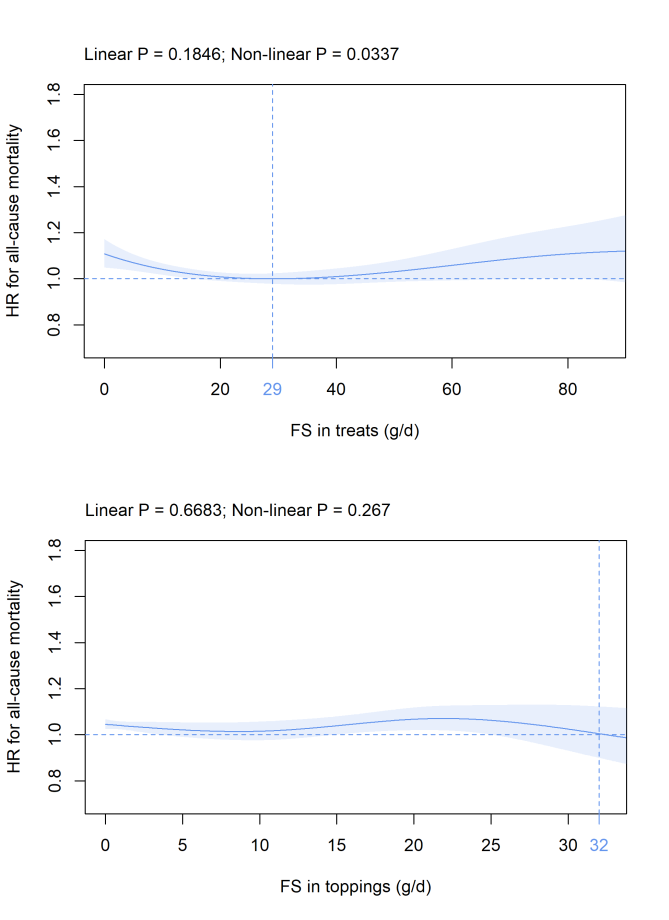


(g) (h)

(i) (j)

(k) (l)

(a) (b)


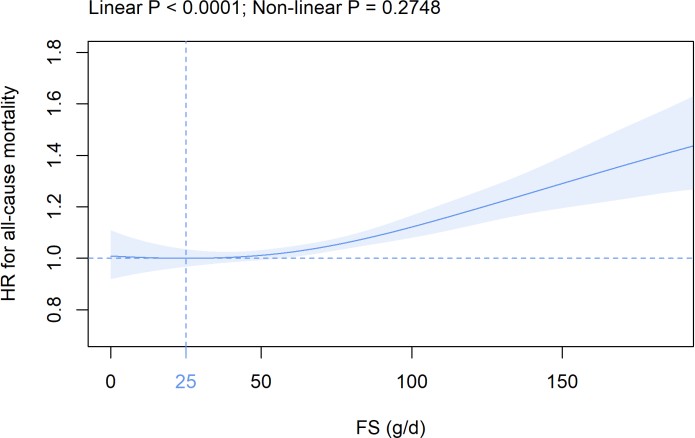

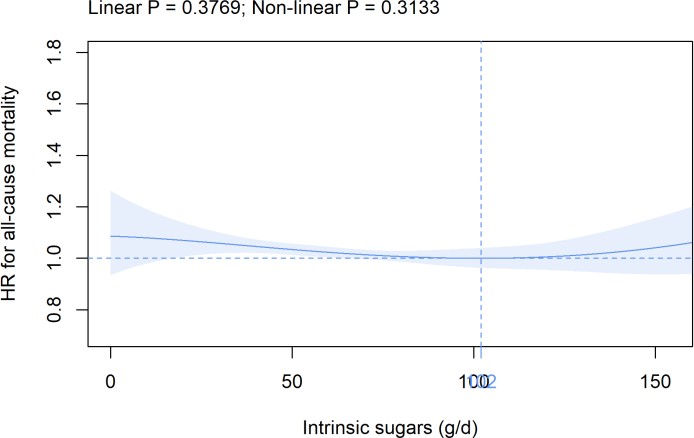


(c) (d)


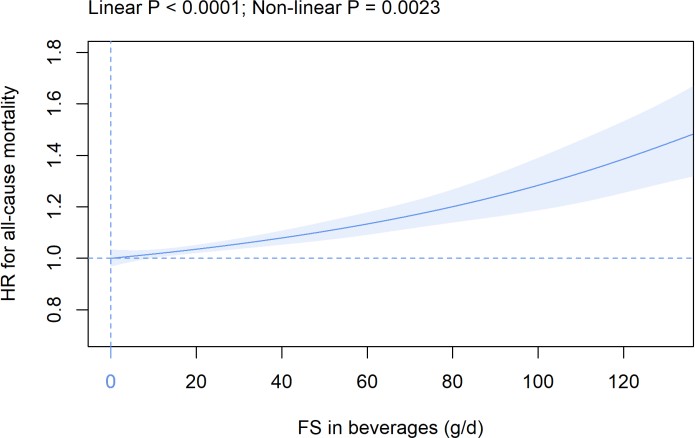

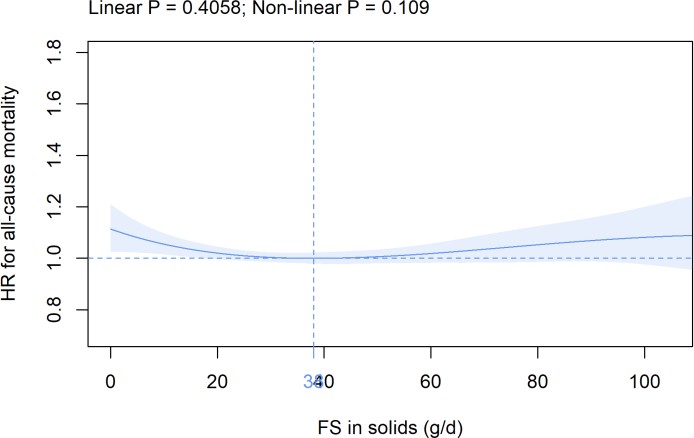


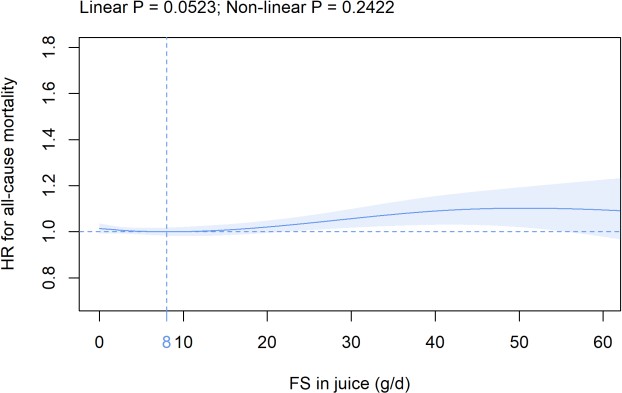
(e) (f)


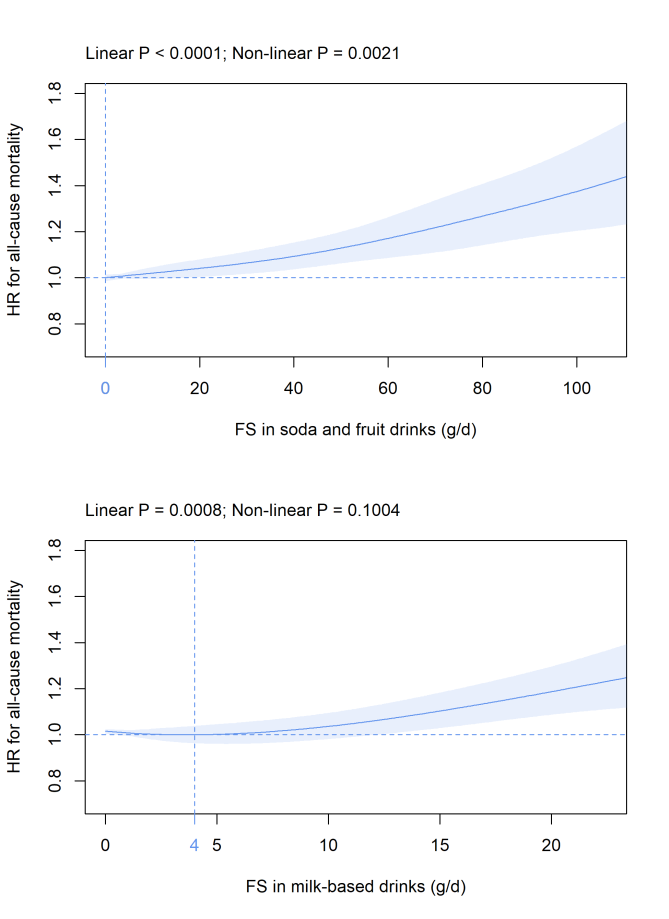

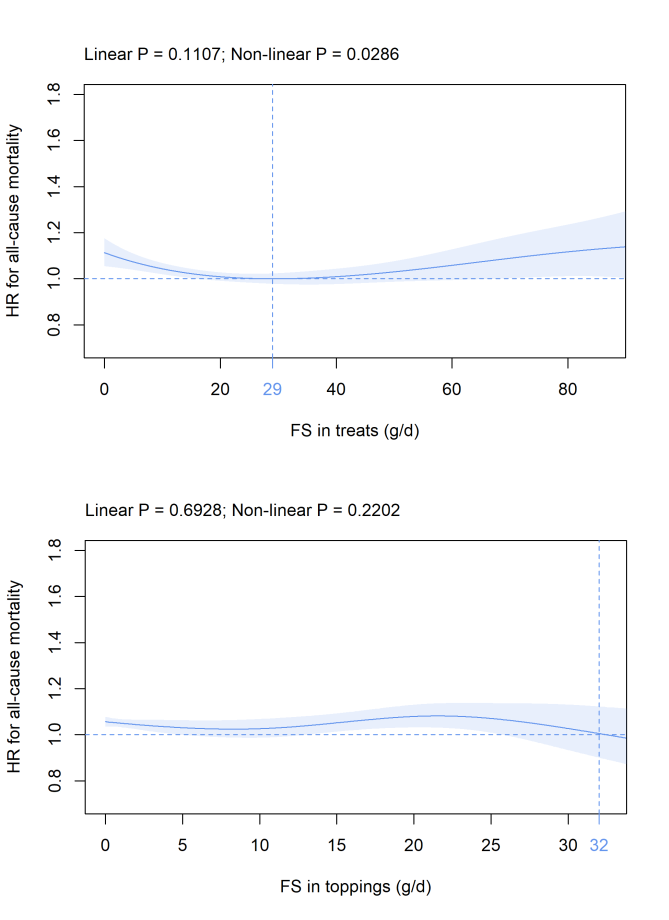


(g) (h)


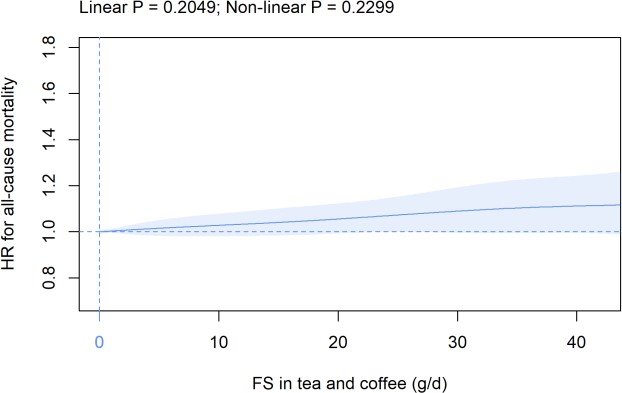


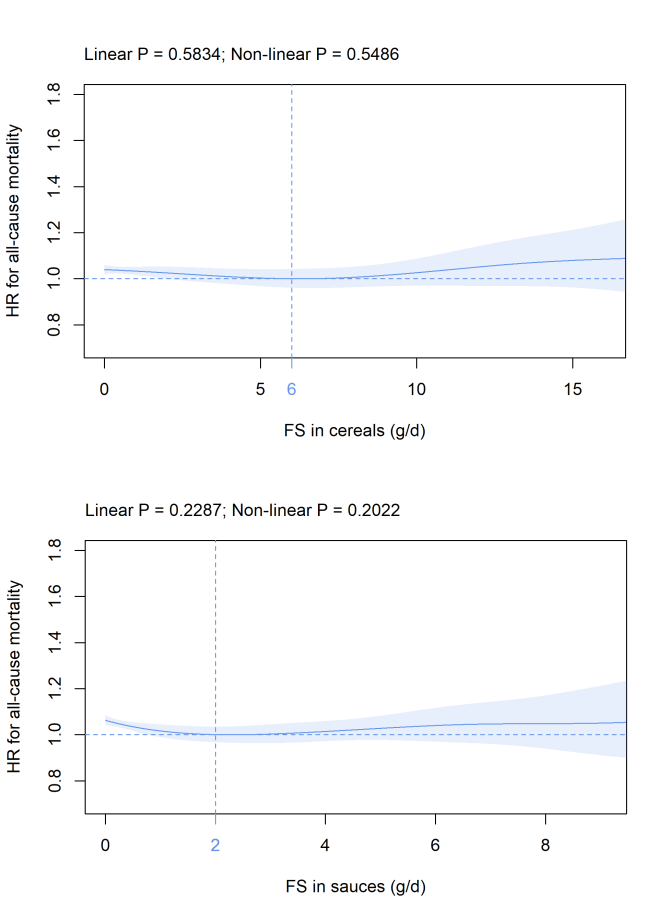
(i) (j)

(k) (l)
